# Reconstructing *Krassilovia mongolica* supports recognition of a new and unusual group of Mesozoic conifers

**DOI:** 10.1371/journal.pone.0226779

**Published:** 2020-01-15

**Authors:** Fabiany Herrera, Gongle Shi, Chris Mays, Niiden Ichinnorov, Masamichi Takahashi, Joseph J. Bevitt, Patrick S. Herendeen, Peter R. Crane

**Affiliations:** 1 Chicago Botanic Garden, Glencoe, Illinois, United States of America; 2 State Key Laboratory of Palaeobiology and Stratigraphy, Nanjing Institute of Geology and Palaeontology and Center for Excellence in Life and Paleoenvironment, Chinese Academy of Sciences, Nanjing, People’s Republic of China; 3 Department of Palaeobiology, Swedish Museum of Natural History, Stockholm, Sweden; 4 School of Earth, Atmosphere and Environment, Monash University, Clayton, Victoria, Australia; 5 Institute of Paleontology and Geology, Mongolian Academy of Sciences, Ulaanbaatar, Mongolia; 6 Department of Environmental Sciences, Faculty of Science, Niigata University, Nishi-ku, Niigata, Japan; 7 Australian Centre for Neutron Scattering, Australian Nuclear Science and Technology Organisation, New South Wales, Australia; 8 Oak Spring Garden Foundation, Upperville, Virginia, United States of America; 9 School of Forestry and Environmental Studies, Yale University, New Haven, Connecticut, United States of America; Baylor University, UNITED STATES

## Abstract

Previously unrecognized anatomical features of the cone scales of the enigmatic Early Cretaceous conifer *Krassilovia mongolica* include the presence of transversely oriented paracytic stomata, which is unusual for all other extinct and extant conifers. Identical stomata are present on co-occurring broad, linear, multiveined leaves assigned to *Podozamites harrisii*, providing evidence that *K*. *mongolica* and *P*. *harrisii* are the seed cones and leaves of the same extinct plant. Phylogenetic analyses of the relationships of the reconstructed *Krassilovia* plant place it in an informal clade that we name the Krassilovia Clade, which also includes *Swedenborgia cryptomerioides–Podozamites schenkii*, and *Cycadocarpidium erdmanni–Podozamites schenkii*. All three of these plants have linear leaves that are relatively broad compared to most living conifers, and that are also multiveined with transversely oriented paracytic stomata. We propose that these may be general features of the Krassilovia Clade. Paracytic stomata, and other features of this new group, recall features of extant and fossil Gnetales, raising questions about the phylogenetic homogeneity of the conifer clade similar to those raised by phylogenetic analyses of molecular data.

## Introduction

Excluding angiosperms, conifers are the most diverse group of living seed plants, with approximately 638 species [1]. Since the taxonomic separation of *Ginkgo* more than a hundred years ago, e.g., [2], conifers have been regarded as a single higher taxonomic unit, e.g., [3, 4] and as a monophyletic group in phylogenetic analyses based on morphological data, e.g., [5–11]. Conifer monophyly was also supported by early phylogenetic analyses of extant plants based on molecular data [12, 13]. Nevertheless, unlike angiosperms, cycads, *Ginkgo* and Gnetales, clear morphological synapomorphies for conifers are difficult to identify, e.g., [6], and some recent phylogenetic studies based on DNA sequence data from large numbers of genes have concluded that conifers may be paraphyletic, e.g., [14–25]. These analyses resolve extant Pinaceae as more closely related to extant Gnetales than to other extant conifers (Araucariaceae, Cupressaceae, Podocarpaceae, *Sciadopitys*, Taxaceae). At the same time, recent paleobotanical data have supported earlier ideas of a close relationship between Gnetales and extinct Bennettitales [26–29], and new discoveries have also identified intriguing similarities in the basic architecture and organization of the ovulate reproductive structures of conifers and some extinct corystosperms [30–32]. These seemingly surprising results highlight the need for a critical reappraisal of the relationships of the diverse fossil plants that are generally regarded as conifers from the Paleozoic and Mesozoic, as a key step toward resolving how living conifers may be related to other groups of extant and extinct seed plants.

Resolving relationships among extinct conifer-like plants is not straightforward because while paleobotanical studies over the past two centuries have described large numbers of potentially relevant fossils, the extent and quality of the information available for these extinct plants is highly variable, e.g., [33–36]. Notwithstanding these difficulties, there have been several important efforts to systematize and analyze the information available for key extinct conifers and to develop models of conifer evolution, e.g., [37–46]. Most significantly, Rothwell et al. [47] developed a matrix to evaluate relationships among many of the better known walchian and voltzialean conifers from the Late Paleozoic and Mesozoic. This analysis was expanded by Escapa et al. [48] who added several Triassic-Jurassic taxa, reconstructed from both seed cones and leaves. An interesting result was the recognition of a clade comprising *Telemachus elongatus*–*Heidiphyllum elongatum*, *Parasciadopitys aequata–Notophytum krauselii* (*Parasciadopitys* now a junior synonym of *Telemachus*; [46]), and *Swedenborgia cryptomerioides–Podozamites schenkii*, all of which have linear, multiveined leaves that are relatively broad compared to those of most living conifers. Herrera et al. [49] added a further taxon to this clade based on their description of *Krassilovia mongolica* from the Early Cretaceous of Mongolia, although at that time the leaves of *Krassilovia* were not known.

In this paper, we present new evidence on the anatomy of the seed cone scales of *Krassilovia*, including the presence of distinctive transversely oriented paracytic stoma that is unusual among conifers, and that help to identify *Podozamites harrisii* as the leaves of the *Krassilovia* plant. This new information further supports recognition of a clade of Mesozoic conifers with multiveined leaves and paracytic stomata, which also includes species of *Cycadocarpidium* and *Swedenborgia* and their associated *Podozamites* leaves. We review the characteristics of this interesting group of putative conifers and assess the potential systematic implications, especially in regard to the hypothesized close relationship between extant Pinaceae and Gnetales based on molecular data.

## Materials and methods

The fossil material examined in this study was isolated from poorly consolidated lignites of the Tevshiingovi Formation at the Tevshiin Govi locality in central Mongolia (45°58’54” N, 106°07’12” E). The Tevshiingovi Formation is considered to be Aptian–Albian in age (125–99.6 Mya) based on stratigraphic correlations [50] and on palynomorphs recovered from the plant localities [51, 52]. Fossil material described in this study is housed in the paleobotanical collections of the Field Museum, Chicago, Illinois (FMNH: collection numbers with the prefix PP) and in the Institute of Paleontology and Geology in Ulaanbaatar, Mongolia (Mongolian Paleontological Center-Flora). A permit obtained for all aspects of this study was granted through a cooperation agreement (A-2019/01) between the Institute of Paleontology and Geology, Mongolian Academy of Sciences, Mongolia (Dr. Khishigjav Tsogtbaatar) and the Chicago Botanic Garden (Dr. Patrick Herendeen).

Bulk lignite samples were disaggregated in water with soap followed by dilute 3% hydrogen peroxide. Organic material was separated from the resulting slurry by gentle sieving and panning over 125–500 μm sieves. The organic material was then air dried and selected mesofossils were cleaned with hydrochloric and hydrofluoric acids, washed thoroughly, and also air dried.

Cuticles from clean leaf and bract-scale complexes were obtained by maceration using dilute household bleach (ca. 1% sodium hypochlorite solution). The thin and delicate cuticles of the leaves and bract-scale complexes required maceration of only a few seconds to less than a minute. Large pieces of cuticle were mounted on slides with glycerin jelly and sealed with nail polish.

Axes, bract-scale complexes and leaves selected for anatomical study were soaked in 10% hydrochloric acid, followed by Aerosol OT (10% solution of sodium dioctyl sulfosuccinate in alcohol), and then taken through an ethanol series (70% to absolute ethanol) before embedding in Technovit 7100 following the prescribed mounting protocol. Transverse and longitudinal sections, ca. 4–7 μm thick, were made of the embedded material using a Leica 2030 microtome. Slides were mounted in Hydromount No. 17966.

Cuticles and anatomical preparations were photographed with differential interference contrast (DIC) illumination using a Leica DMLB microscope at the Chicago Botanic Garden. Cuticles for scanning electron microscope (SEM) examination were mounted on stubs with conductive tape, coated with gold, and imaged using a Carl Zeiss EVO 60 scanning electron microscope at the Field Museum, Chicago.

Leaf mass per area (M_a_) was estimated using methods described by Royer et al. [53] and the equation derived for broadleaf gymnosperms [54, 55], based on 73 complete to nearly complete leaves of *Podozamites harrisii* for which leaf area could be reasonably estimated by digital reconstruction with ImageJ.

Two isolated bract-scale complexes were analyzed to explore the spatial distribution of sclerified tissues using thermal-neutron tomography at the DINGO tomographic station at the Open-Pool Australian Lightwater (OPAL) reactor at the Australia Nuclear Science and Technology Organisation (ANSTO), Lucas Heights, New South Wales, Australia (see Mays et al. [56] for additional information on required settings). Neutron tomographs were reconstructed from a compilation of 600 evenly-spaced projections across a total rotation of 180°. Each projection consisted of four accumulations, each of 10 second exposure length. The pixel width for these projections was approximately 12.97μm. The image capture was with an Andor IKON-L CCD camera (liquid cooled, 16-bit). Tomographic reconstructions were performed using Octopus Reconstruction v.8.8 (Inside Matters NV), and volume rendering and visualizations were performed using Avizo v.9.0.1 (FEI Company).

To assess the phylogenetic position of the *Krassilovia* plant and related fossils we used a morphological matrix for Late Paleozoic and Early Mesozoic conifers modified from that developed by Rothwell et al. [47] as expanded by Escapa et al. [48] and Herrera et al. [49]. Here, we also add *Pseudovoltzia liebeana* [42, 57]; *Manifera talaris* [58], *Emporia lockardii* [59], *Emporia cryptica* [60], and *Emporia royalii* [61] to the morphological matrix (S1 Appendix; Table 1).

**Table 1:** List of Late Paleozoic to Early Mesozoic and living conifers included in the core phylogenetic analyses, and also the gnetalean plant *Dechellyia*-*Masculostrobu*s.

Taxon	Age*	Ocurrence	References
† *Aethophyllum stipulare*	ET–MT	Grès à Voltzia Delta, France	[90, 91]
† *Barthelia furcata*	LPv–EP	Hamilton Quarry, Kansas, USA	[92]
† *Callistophyton poroxyloides*	LPv	North America	[93–96]
*Cedrus deodara*	Living	Asia	[72]
† *Concholepis harrisii*	EP–MP	Western Angaraland, Russia	[44]
† *Cordaixylon dumusum*	LPv	North America	[97, 98]
*Cunninghamia lanceolata*	Living	Asia	[72]
† *Cycadocarpidium erdmanni-Podozamites schenkii*	LT	Iran	[33, 62, 99]
† *Dechellyia gormanii-Masculostrobus clathratus*	LT	Arizona, North America	[66]
† *Dicranophyllum hallei*	EP	Europe	[100, 101]
† *Dolomitia cittertiae*	LP	Alps, Italy	[42]
† *Elatides zhoui*	EC	Mongolia	[64]
† *Emporia cryptica*	LPv	Hamilton Quarry, Kansas, USA	[60]
† *Emporia lockardii*	LPv	Hamilton Quarry, Kansas, USA	[59]
† *Emporia royalii*	LPv	Hamilton Quarry, Kansas, USA	[61]
† *Ernestiodendron flliciforme*	LPv–EP	Europe	[37]
† *Ferugliocladus spp*.	MP	Argentina	[102]
† *Genoites patagonica*	MP	Argentina	[103]
† *Hanskerpia hamiltonensis*	LPv	Hamilton Quarry, Kansas, USA	[47]
† *Krassilovia mongolica-Podozamites harrisii*	EC	Mongolia	[49, 63], this study
† *Kungurodendron sharovii*	EP–MP	Western Angaraland, Russia	[44]
† *Majonica alpina*	LP	Alps, Italy	[42]
† *Manifera talaris*	EP	Texas, USA	[58]
† *Mesoxylon priapii*	LPv	North America	[104]
† *Ortiseia spp*.	LP	Alps, Italy	[105, 106]
† *Otovicia hypnoides*	LPv–EP	Germany	[107]
† *Pseudovoltzia liebeana*	LP	Europe	[57, 108]
† *Schizolepidopsis canicularis*	EC	Mongolia	[65]
*Sciadopitys verticillata*	Living	Japan	[72]
† *Swedenborgia cryptomerioides-Podozamites schenkii*	LT–EJ	Europe	[33, 67]
† *Telemachus elongatus- Heidiphyllum elongatum*	MT–LT	Antarctica	[46, 48, 109]
† *Thucydia mahoningensis*	LPv–EP	Ohio, North America	[110, 111]
† *Timanostrobus muravievii*	EP–MP	Western Angaraland, Russia	[44]
† *Utrechtia floriniformis*	LPv–EP	Europe	[37, 112, 113]
† *Vojnovskyean plant*	LPv–EP	North America	[114]
† *Voltzia hexagona*	EP	Europe	[115]

*Pv, Pennsylvanian; P, Permian; T, Triassic; J, Jurassic; K, Cretaceous; E, Early; M, Middle; L, Late.

^†^Fossil Taxa.

Following Escapa et al. [48], *Telemachus elongatus* (ovulate cone)-*Heidiphyllum elongatum* (leaf), and *Swedenborgia cryptomerioides* (ovulate cone)-*Podozamites schenkii* (leaf) are included as single terminals in our phylogenetic analyses. *Parasciadopitys aequata* (ovulate cone)-*Notophytum krauselii* (axes and leaves) are not included since these fossils are now recognized as the permineralized states of the *Telemachus* plant [46]. We added the terminal *Cycadocarpidium erdmanni* (ovulate cone)-*Podozamites schenkii* (leaf) based on the illustration of a seed cone of *Cycadocarpidium erdmanni* from the Late Triassic of Iran that is borne terminally on a shoot bearing narrow leaves of *P*. *schenkii* ([62]; S2 Appendix). *Krassilovia mongolica*, included in the phylogenetic analysis of Herrera et al. [49], is treated here as the terminal *Krassilovia mongolica* (ovulate cone)-*Podozamites harrisii* (leaf) based on their co-occurrence at Tevshiin Govi and similarities in their cuticles (see below; [63]; S3 Appendix).

We further expanded taxonomic coverage of the analysis by including an early fossil member of the Cupressaceae (*Elatides zhoui;* [64]) as a placeholder for the Araucariaceae-Cupressaceae-Podocarpaceae-*Sciadopitys*-Taxaceae clade of extant conifers, and *Schizolepidopsis canicularis* [65] as a placeholder for extant Pinaceae. We also added extant genera as placeholders for three extant families: *Cunninghamia lanceolata* for Cupressaceae, *Cedrus deodara* for Pinaceae and *Sciadopitys verticillata* for Sciadopityaceae. We also assessed the position of the *Dechellyia gormanii*-*Masculostrobus clathratus* plant [66], a putative early gnetalean [7], in relation to the other fossils and extant plants that we considered (S4 Appendix).

Most of the characters and their scoring are based on Rothwell et al. [47], but several characters are redefined and modified from the original definitions [47] and from Escapa et al. [48]. Changes in scoring are noted in the descriptions of the characters (S1 Appendix). Scoring of *Swedenborgia* is as per Escapa et al. [48], except where noted. Scoring of the *Telemachus* plant is based on Escapa et al. [48] and Bomfleur et al. [46]. Scoring of *Krassilovia* is as per Herrera et al. [49], except where noted and with the addition of information on the leaves. Scoring of *Cycadocarpidium* is based on the descriptions in Harris [33, 67] and Schweitzer & Kirchner [62]. Scoring of *Pseudovoltzia liebeana* is based on Clement-Westerhof [42] and Schweitzer [57]. Scoring of *Manifera talaris* is based on Looy and Stevenson [58]. Scoring of the *Dechellyia gormanii*-*Masculostrobus clathratus* plant is based on Ash [66] and observations of the type material. The complete modified morphological matrix, which includes 52 morphological characters (S1 Appendix) and 39 taxa (including *Callistophyton*), as well as all revised definitions and scoring, is available at the MorphoBank website (http://www.morphobank.org; project 3184).

Previous studies of Paleozoic and Mesozoic conifers [47–49] rooted their analysis on *Callistophyton*. We scored *Callistophyton* in our matrix, and included it in our previous analysis (49), but we regard it as inapplicable for many of the characters scored in this analysis because of its very different fern-like leaves and ovulate and pollen structures compared to those of coniferophytes. Therefore, for current purposes we exclude *Callistophyton* and root the analysis on the two cordaitaleans, *Cordaixylon* and *Mesoxylon*, resolved as the sister group to conifers in previous analyses [47–49].

Our core analysis included 34 fossil and living taxa and 51 morphological characters. We then conducted a simple experiment to explore the possible position of extant Gnetales by adding the putative early gnetalean plant *Dechellyia gormanii*-*Masculostrobus clathratus* (S4 Appendix). We also experimented with the inclusion of two representatives of Cheirolepidiaceae and *Lebowskia grandifolia*, an additional Permian conifer (S5 Appendix). Parsimony analyses and heuristic searches were carried out with 10000 replicates of random taxon addition and tree-bisection-reconnection (TBR) branch swapping in the program PAUP* 4.0a (build 165) for Macintosh (X86) [68]. All morphological characters were treated as unordered. Bootstrap analysis was performed on the morphological data using 100 replicates and full heuristic searches.

## Results

### Cuticular anatomy of the seed cone scales

**Fig 1:**
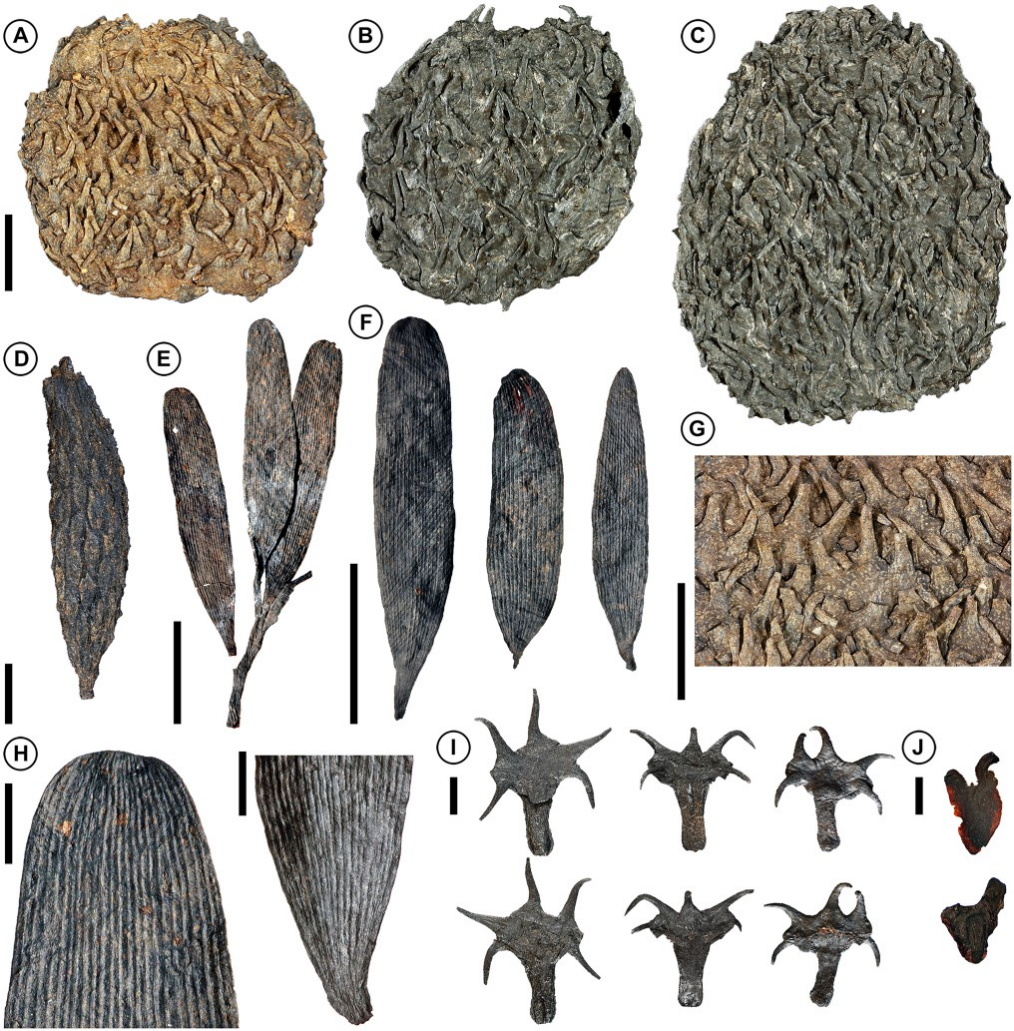
Seed cones, cone axis, bract-scale complexes, and winged seeds of *Krassilovia mongolica* and associated leaves of *Podozamites harrisii*. (A–C) Articulated seed cones showing tightly imbricate interlocking bract-scale complexes (A: PP55848; B: PP59064; C: PP59065). (D) Isolated cone axis; note conspicuous spirally arranged abscission scars (PP59066). (E) Incomplete leafy shoot showing a cluster of three attached leaves (one represented only by the leaf base); the fourth leaf (left) was attached to the axis when discovered (PP56218). (F) Three detached strap-shaped leaves; note variation in leaf size and shape, and conspicuous parallel venation (PP56226; PP56223; PP56222). (G) Detail of A showing tightly imbricate interlocking bract-scale complexes. (H) Detail of leaf apex showing converging veins (left; PP56228); leaf base showing the absence of a clearly differentiated petiole (right; PP56230). (I) Three isolated bract-scale complexes showing abaxial (top) and adaxial (bottom) surfaces; note three prominent, spiny, distal lobes and two prominent, spiny, proximal lobes (PP59067; PP59068; PP59069). (J) Two isolated seeds showing narrow wings and variation from more or less symmetrical (top; PP59070), to strongly asymmetrical (bottom; PP59071). Scale bars: E, F = 1 cm; A–C, G = 5 mm; D, H, I = 2 mm; J = 1 mm.

**Fig 2:**
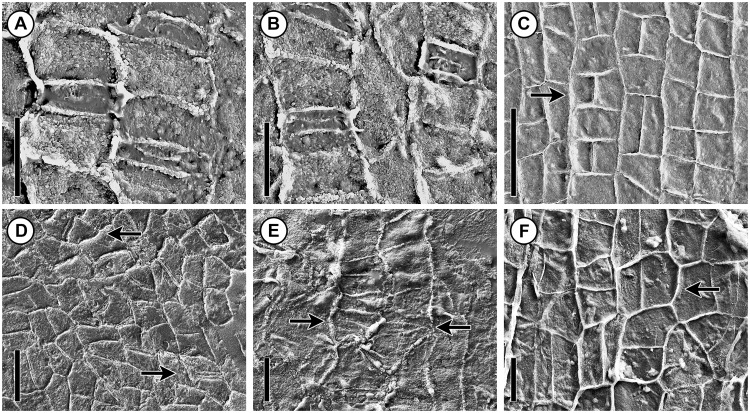
Scanning electron micrographs showing similarities between inner surface of cuticles of leaves of *Podozamites harrisii* (A–C) and bract-scale complexes of *Krassilovia mongolica* (D–F). (A, B) Detail of stomatal band from abaxial leaf cuticle showing cell outlines of transversely oriented, paracytic (monocyclic) stomata (PP56233). (C) Detail from adaxial leaf cuticle showing rectangular epidermal cell outlines formed by regular transverse and longitudinal divisions; note that many epidermal cell outlines are arranged in pairs (arrow) (PP56234). (D, E) Cuticle from bract-scale complexes showing cell outlines of paracytic (monocyclic) stomata (arrows) that are transversely oriented relative to the files of epidermal cells (D: PP59072; E: PP56235). (F) Cuticle from bract-scale complex showing rectangular epidermal cell outlines formed by regular transverse and longitudinal divisions; note that many epidermal cell outlines are arranged in pairs (arrow) (PP56236). Scale bars: C = 50 μm; A, B, D–F = 20 μm.

**Fig 3:**
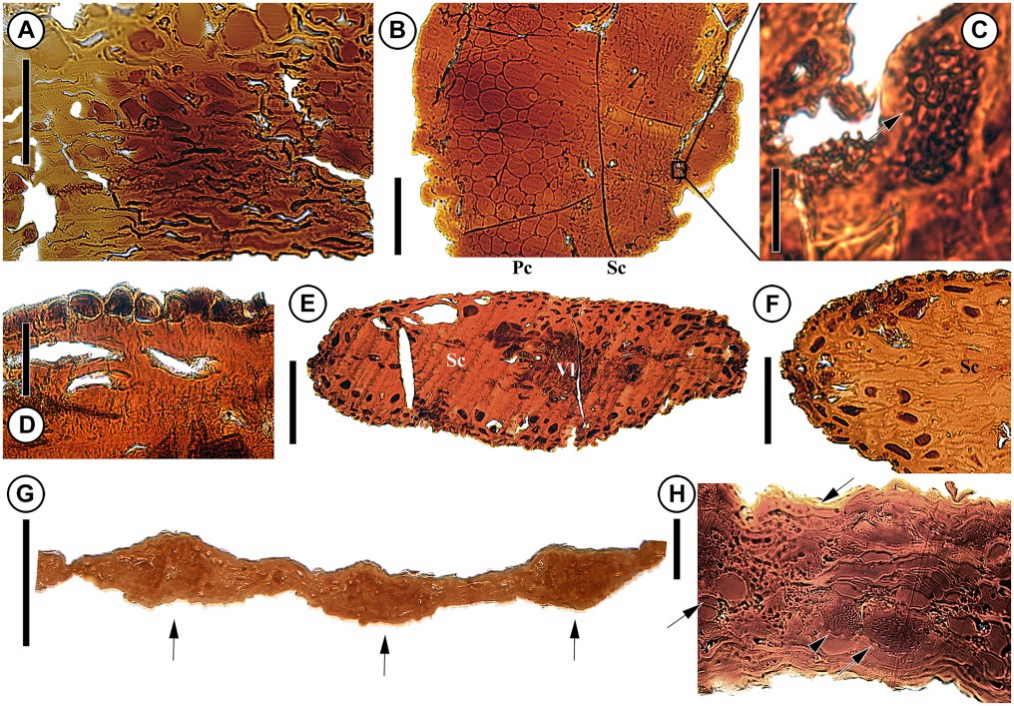
Light micrographs showing anatomy of axis and bract–scale complexes of *Krassilovia mongolica* (A–F) and leaves of *Podozamites harrisii* (G, H). (A) Transverse section of the axis showing collapsed thin-walled cells with scattered sclerenchyma cells (PP59073). (B) Detail of transverse section of a bract-scale complex near the base of the stalk; note sclerenchyma (Sc) and well developed parenchyma (Pc) (PP59074). (C) Detail from B inside Sc showing an extremely small amount of xylem; note xylem with relatively thin walls and wide lumens (arrow). (D) Detail of transverse section of bract-scale complex showing thin cuticle and well developed epidermis; note the dark contents of the epidermal cells (PP59074). (E) Transverse section through a distal lobe of a bract-scale complex showing central vascular tissue with xylem (Vl) surrounded by sclerenchyma (PP59074). (F) Detail of transverse section of bract-scale complex showing well-developed sclerenchyma (Sc) and scattered cells with dark contents (PP59074). (G) Transverse section of leaf showing the position of three vascular bundles (arrows) (PP59075). (H) Detail of transverse section of leaf showing thin cuticle (top arrow), large thin-walled mesophyll cells with dark contents (middle arrow), xylem cells (arrowhead) flanked by large patches of transfusion tracheids-like cells (lower arrow) (PP59075). Scale bars: B, E, G = 200 μm; A, F = 100 μm; D, H = 50 μm; C = 40 μm.

The lignified seed cone scales of *Krassilovia mongolica* from Tevshiin Govi are abundant and very well preserved [49] (Fig 1). Nevertheless, their cuticles are extremely thin and delicate. The cuticles are fragile to manipulate and difficult to isolate. Abaxial and adaxial cuticles are ca. 0.7–1.2 μm thick (Figs 2 and 3). Epidermal cell outlines are usually isodiametric (nearly square-shaped) or rectangular in outline ca. 14–31 μm in diameter. The pattern of cell outlines often shows evidence of relatively late transverse and longitudinal divisions resulting in the epidermal cells often being arranged in pairs (Fig 2D–2F). In many cases these pairs of cells tend to be perpendicular to each other (Fig 2D–2F), sometimes creating a distinctive quartet of four cells.

Stomata are very sparse (Fig 2D and 2E). Each stomatal complex is more or less rectangular in outline, monocyclic, and consists of the outlines of two guard cells flanked by the outlines of two lateral subsidiary cells (paracytic). Stomatal complexes are transversely oriented relative to the longitudinal files of epidermal cells (Fig 2E). Each stomatal complex is 10–21 μm long (distance between the polar walls of the guard cells) and 17–24 μm wide (distance between the outermost walls of the lateral subsidiary cells).

Guard cells are narrowly and regularly rectangular in outline, not sunken, and with straight anticlinal walls (Fig 2D and 2E). The stomatal aperture is slit-like. Lateral subsidiary cells are symmetrical or asymmetrical, more or less rectangular in outline, oriented parallel to the stomatal aperture and extend for the whole length of the guard cells (Fig 2D and 2E). Subsidiary cells are similarly cutinized to ordinary epidermal cells with a comparable fine granular inner cuticular surface (Fig 2D and 2E). The outer surface of the cuticle over the guard cells and subsidiary cells is smooth and lacks cuticular thickenings or papillae.

### Linking *Krassilovia mongolica* and *Podozamites harrisii*

Several lines of evidence suggest that the plants that produced *Krassilovia mongolica* cones bore strap-shaped, elongate, and multiveined *Podozamites* leaves. In the Tevshiin Govi lignite, which is interpreted as a predominantly autochthonous swamp deposit, articulated seed cones are relatively rare but isolated cone axes, bract-scale complexes, and seeds of *K*. *mongolica* are exceptionally abundant [49], as are shoots and leaves of *P*. *harrisii* (Fig 1; S3 Appendix; [63]). A similar association also occurs in new collections of compression/impression fossils made at the approximately coeval Shine Khudag locality (GPS: 44°43'2.60"N; 107°55'39.0"E), ca. 210 Km southeast of Tevshiin Govi (see also [69]) (S6 Appendix). The Shine Khudag plant fossils were preserved in a non-swamp, possibly lacustrine, depositional environment, but as at Tevshiin Govi, the flora is rich in articulated and disarticulated *Podozamites* shoots and leaves, as well as isolated *Krassilovia* cone scales and winged seeds.

In addition to field association, there are strong similarities between the epidermal features of *K*. *mongolica* bract-scale complexes and the leaves of *P*. *harrisii* (Fig 2) [63]. Both species have extremely thin, delicate cuticles, as well as epidermal cell outlines that are often square to rectangular in outline and show evidence of late transverse and longitudinal divisions. Outlines of epidermal cells frequently occur in a paired arrangement (Fig 2C and 2F). Most significantly, the stomatal complexes of both organs are about the same size (~16–28 μm long and 22–40 μm wide), transversely oriented, paracytic and monocyclic. The outlines of the guard cells are not sunken, and are flanked by the outlines of two lateral subsidiary cells (Fig 2A, 2B, 2D and 2E).

No other leaf or reproductive structure recovered from the Tevshiin Govi locality has the characteristic transversely oriented, paracytic stomata and paired epidermal cell outlines seen in the bract-scale complexes of *K*. *mongolica* and leaves of *P*. *harrisii*. Two other strap-shaped, multiveined leaves at Tevshiin Govi, assigned to *Pseudotorellia resinosa*, and *P*. *palustris* [63], have fewer veins (4–14 vs. 14–25 per leaf), thick cuticles, and longitudinally oriented stomata in which the guard cells are sunken and surrounded by 2–5 lateral subsidiary cells, and 1–3 polar cells that resemble normal epidermal cells [63]. *Pseudotorellia resinosa* is the leaf of the ginkgophyte *Umaltolepis mongoliensis* [70], while *P*. *palustris* is inferred to be the leaf of the corystosperm *Umkomasia mongolica* [31].

### Anatomy of *Podozamites harrisii* leaves and *Krassilovia mongolica* bract-scale complexes

Leaves of *Podozamites harrisii* are strap-shaped, multiveined (14–25 conspicuous, longitudinal veins) and are borne helically on slender shoots that show only small persistent leaf cushions [63]. Leaves on a shoot are flattened into a single plane by twisting of their bases, as in many extant conifers [71, 72]. Leaf mass per area (M_a_), estimated from leaf laminas and petiole widths of isolated, intact to nearly intact *P*. *harrisii* leaves, is 207.45 g/m^2^ [95% prediction interval = 178.88; 234.22].

*Podozamites harrisii* leaves are hypostomatic with a thin, delicate cuticle on both the adaxial and abaxial leaf surfaces [63]. Transverse sections show a thin cuticle with an epidermis, hypodermis, large thin-walled mesophyll cells with dark contents, and scattered sclereids. The xylem cells comprising the vascular bundles are associated with large concentrations of cells resembling transfusion tracheids (Fig 3G and 3H).

**Fig 4:**
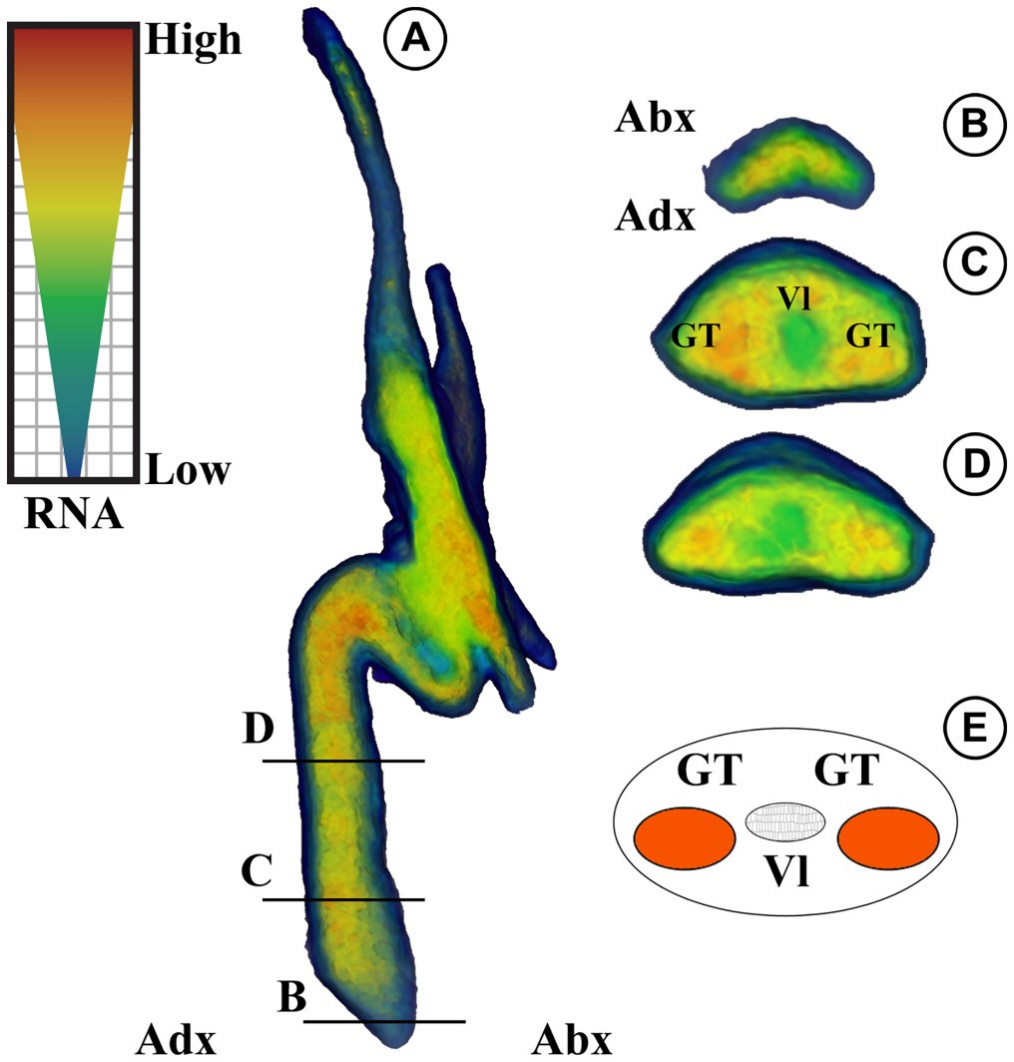
Neutron tomographic reconstruction of isolated bract–scale complex of *Krassilovia mongolica* (PP59076). RNA = Relative Neutron Attenuation (high to low), cross-hatch area represents relative transparency (top left box). (A) Volume rendering of lateral view of bract-scale complex; (Abx, abaxial: Adx, adaxial). (B–D) Transverse sections from base to top of stalk for the bract-scale complexes in (A). Epidermis and hypodermis has lowest neutron attenuation (blue); vasculature tissue (Vl) has the intermediate neutron attenuation (green); cortical ground tissues (Gt) have the highest neutron attenuation. (E) Diagrammatic reconstruction near the base of stalks; vasculature appears slightly close to the abaxial (Abx) and the ground tissue forms two strands near the adaxial side (Adx) (see also Fig 3B and 3C and S7 Appendix).

Sections of *Krassilovia mongolica* bract-scale complexes show an extremely thin cuticle and a distinct epidermal layer with dark contents (Fig 3D). Near the base, stalks of the bract-scale complexes are composed mostly of ground tissue (sclerenchyma and large parenchyma cells) and very few relatively thin-walled xylem with wide lumens (Fig 3B and 3C). The single vascular strand is slightly displaced abaxially, divides distally and extends into each of the five spiny lobes. The ground tissue is arranged in two elongated zones adjacent to the vascular strand on the adaxial side (Figs 3 and 4; S7 Appendix). Distally the bract-scale complexes contain large amounts of sclerified tissue and xylem (Fig 3E and 3F).

### Phylogenetic relationships of the *Krassilovia*–*Podozamites* plant

**Fig 5:**
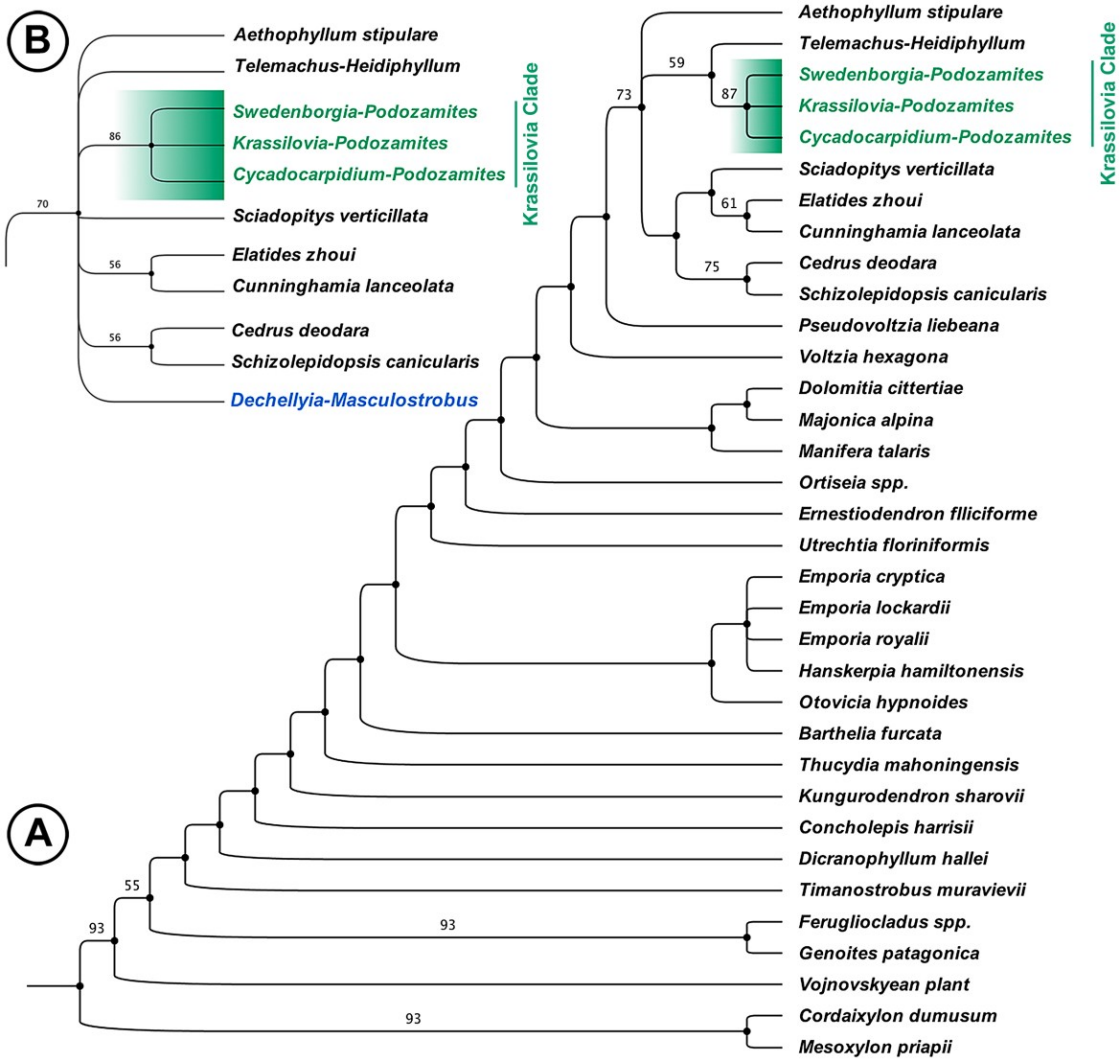
Phylogenetic analyses of selected Late Paleozoic to Early Mesozoic conifers. (A) Strict consensus of 10 most parsimonious trees of 190 steps showing the Krassilovia Clade with *Swedenborgia*-*Podozamites*, *Cycadocarpidium*-*Podozamites*, and the *Krassilovia* plant. (B) Detail of strict consensus of 41 most parsimonious trees of 198 steps with the inclusion of the *Dechellyia*-*Masculostrobus* plant, a putative early gnetalean (see S5 Appendix for complete tree). Bootstrap values given for nodes > 50%.

Our core phylogenetic analysis (Fig 5A), with cordaitaleans (*Cordaixylon* and *Mesoxylon*) as the outgroup and all fossils (except the *Dechellyia*-*Masculostrobus* plant and Cheirolepidiaceae), including early crown Cupressaceae (*Elatides*), probable stem Pinaceae (*Schizolepidopsis*), and extant conifers (*Sciadopitys*, *Cedrus*, *Cunninghamia*) resulted in 10 most parsimonious trees (length 190 steps, consistency index [CI] 0.411, retention index [RI] 0.715, and rescaled consistency index [RC] 0.294). This analysis yielded the smallest number of most parsimonious trees and greatest phylogenetic resolution when compared with other simple experiments (e.g., excluding living conifers, excluding *Elatides* and *Schizolepidopsis*, including Cheirolepidiaceae; S5 Appendix). The strict consensus tree (Fig 5A) is relatively resolved and suggests relationships among late Paleozoic and early Mesozoic conifers more or less similar to those of previous analyses [47–49], for example, the clade [*Genoites* + *Ferugliocladus*] is conserved for these South American Gondwanan taxa.

The consensus tree (excluding the *Dechellyia*-*Masculostrobus* plant and Cheirolepidiaceae) suggests further resolution and relatively high bootstrap values for crown conifer groups in our analysis (Fig 5A). In the strict consensus tree and in all experiments conducted (see also S5 Appendix), the *Krassilovia*-*Podozamites* plant is resolved as part of a clade comprising [*Cycadocarpidium* + *Swedenborgia* + *Krassilovia*], with *Telemachus* as its sister group. We refer to this group of three taxa [*Krassilovia* + *Cycadocarpidium* + *Swedenborgia*] as the Krassilovia Clade, which we diagnose as a clade of conifers, or conifer-like plants, characterized by the combination of strap-shaped distichous leaves (arranged in one plane in two ranks on opposite sides of the axis) with transversely-oriented paracytic stomata (i.e., two lateral subsidiary cells parallel to the guard cells), and seed cones with bract-scale complexes of variable form. The strap-shaped leaves of the Krassilovia Clade (*Cycadocarpidium*, *Krassilovia*, *Swedenborgia*) are all of the *Podozamites* type, whereas the sister taxon *Telemachus* has strap-shaped leaves of *Heidiphyllum* type. Anderson and Anderson [73] noted that *Heidiphyllum* may be associated with both *Telemachus* (probable conifer) and *Dordrechtites* (possible corystosperm). *Podozamites* and *Heidiphyllum* leaves both have a large number of parallel veins, e.g., [33, 46, 62, 63, 74], but stomata of some *Heidiphyllum* leaves are longitudinally oriented, with 5–6 subsidiary cells, and papillae around the stomatal pits [73]. Paracytic stomata are not present in *Heidiphyllum* and the leaves were likely borne spirally on short shoots [46]. Notwithstanding these potential differences, which may arise from uncertainty about the cuticular anatomy of different kinds of *Heidiphyllum* leaves, we hypothesize that *Telemachus* is closely related to the Krassilovia Clade because of similarities to *Cycadocarpidium*, *Krassilovia*, and *Swedenborgia* in the shape and form of the bract-scale complexes (see [49]).

The Krassilovia Clade, plus the *Telemachus*-*Heidiphyllum* plant, comprise one branch of a trichotomy with the putatively herbaceous Triassic conifer *Aethophyllum*, and a clade comprising living and fossil conifers, in which Pinaceae [*Cedrus* + *Schizolepidopsis*] are the sister group to *Sciadopitys* plus living and fossil Cupressaceae [*Cunninghamia* + *Elatides*] (Fig 5A).

Adding the *Dechellyia*-*Masculostrobus* plant, a putatively early gnetalean (S4 and S5 Appendices), to the core analysis resulted in 41 most parsimonious trees (length 198 steps, CI: 0.399, RI: 0.708, and RC: 0.283) and slightly lower bootstrap values (Fig 5B). Most relationships remained the same, but the *Dechellyia*-*Masculostrobus* plant was resolved in a polytomy with the Krassilovia Clade [*Krassilovia* + *Cycadocarpidium* + *Swedenborgia*], *Aethophyllum*, *Telemachus*, [*Cedrus* + *Schizolepidopsis*], [*Elatides* + *Cunninghamia*], and *Sciadopitys* (Fig 5B).

Further experiments with adding two members of the Cheirolepidiaceae and an additional Permian conifer (S5) to the core analysis along with the *Dechellyia*-*Masculostrobus* plant resulted in 48 most parsimonious trees (length 204 steps, CI: 0.392, RI: 0.712, and RC: 0.279). Resolution was substantially reduced at the base of the tree, but a group of extinct and extant conifers was still recovered with *Aethophyllum* and *Telemachus* forming successive sister-groups to a group composed of the Krassilovia Clade plus a clade composed of ([*Elatides* + *Cunninghamia*] + (*Schizolepidopsis* (*Cedrus* (*Dechellyia*-*Masculostrobus* (*Sciadopitys* + Cheirolepidiaceae))))).

### Systematics and nomenclature

Class: Coniferopsida

Order: Voltziales

**Fig 6:**
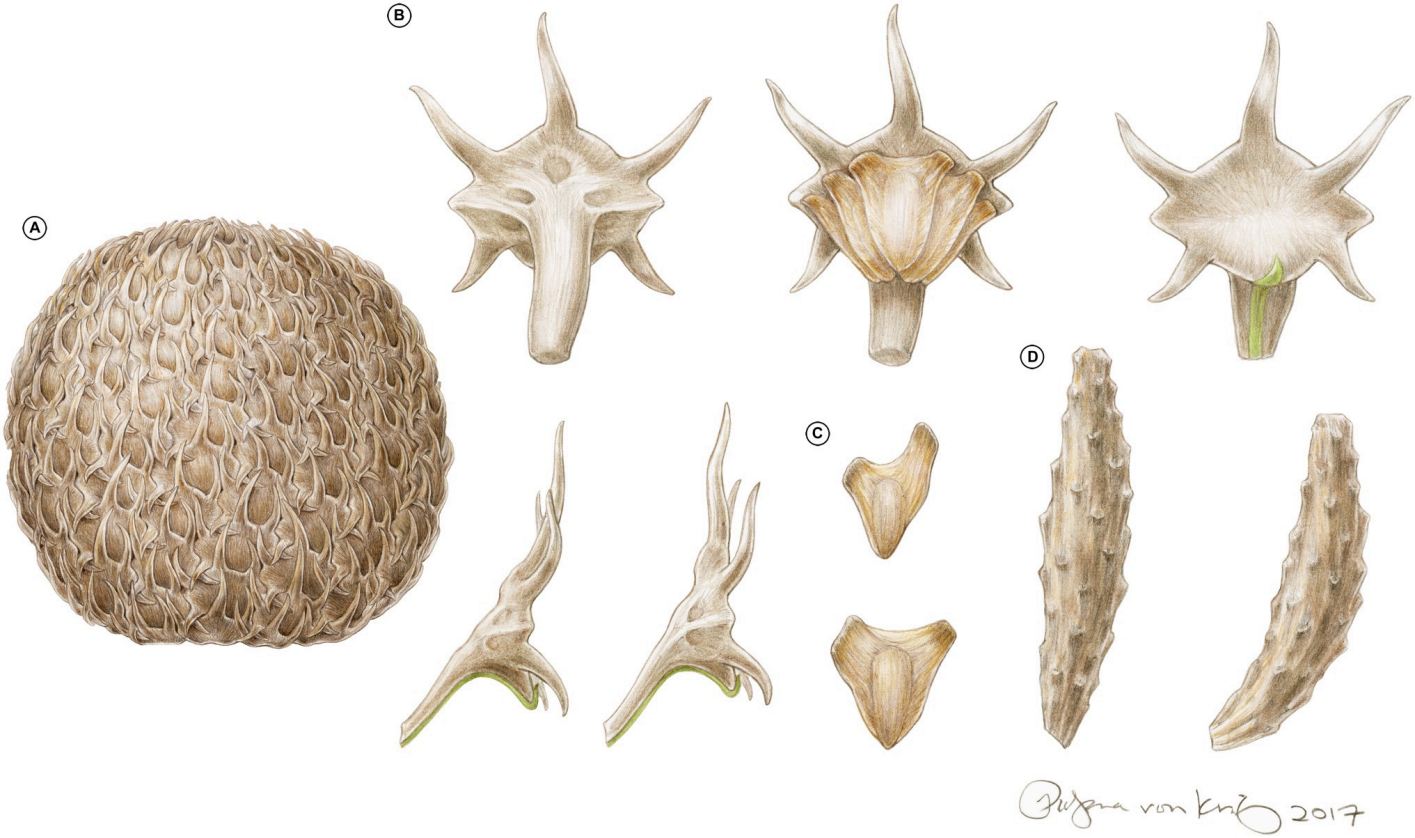
Reconstruction of *Krassilovia mongolica*. (A) Complete mature seed cone showing the strongly imbricate spiny bract-scale complexes. (B) Isolated bract-scale complex in adaxial view showing five seed scars (top left), isolated bract-scale complex in adaxial view with five seeds (top middle), isolated bract-scale complex in abaxial view showing the inconspicuous leafy bract (top right); isolated bract-scale complexes in lateral view showing seed scars and leafy bract (bottom). (C) Isolated asymmetrical (top) more or less symmetrical (bottom) winged seeds. (D) Isolated seed cone axes showing prominent abscission scars. Drawings not to scale. Credit: Pollyanna von Knorring.

**Fig 7:**
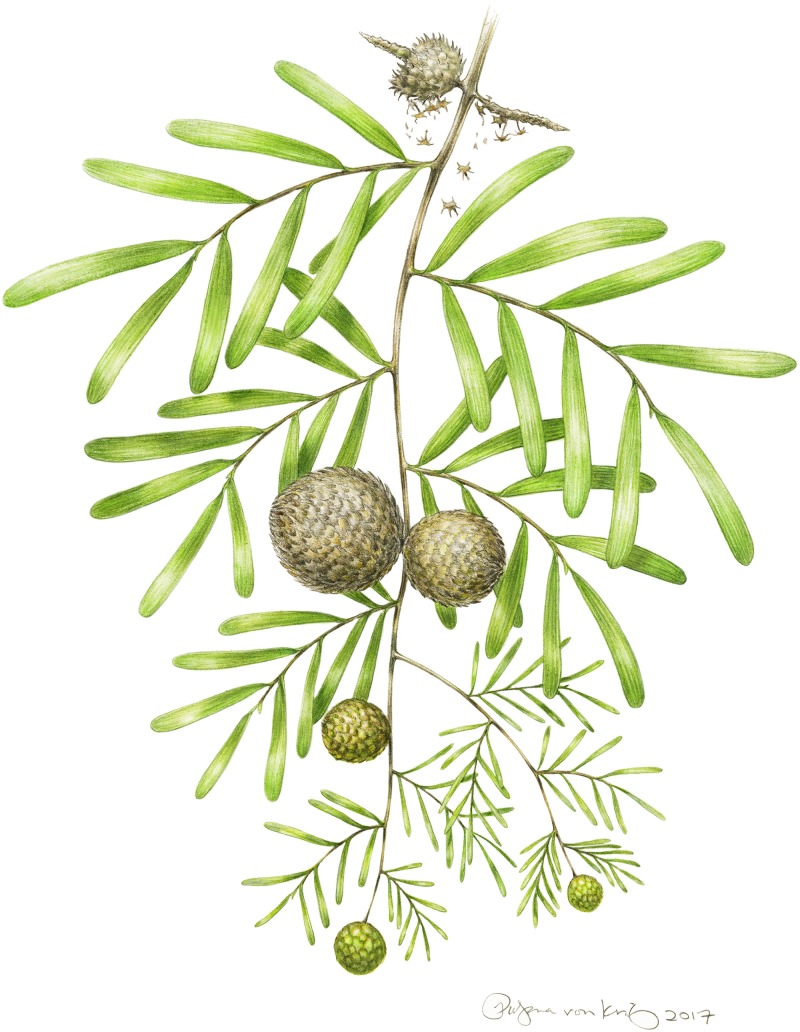
Reconstruction of a branch of *Krassilovia mongolica* bearing terminal seed cones and alternately arranged leafy shoots of *Podozamites harrisii*. Mature and maturing cones are depicted distally showing the ultimate disarticulation of the bract-scale complexes and the dispersal of the winged seeds. Credit: Pollyanna von Knorring.

Family: Krassiloviaceae Herrera et al. fam. nov. Type: *Krassilovia* Herrera, Shi, Leslie, Knopf, Ichinnorov, Takahashi, Crane et Herendeen. Int. J. Plant Sci. 176:793, 2015. *Krassilovia mongolica* Herrera, Shi, Leslie, Knopf, Ichinnorov, Takahashi, Crane et Herendeen. (Figs 1–4 and 6–7).

Familial diagnosis: Leaves distichously arranged on slender deciduous shoots, borne helically on small persistent leaf cushions, but flattened into a single plane by twisting of their bases. Leaves narrowly oblong to strap-shaped, with multiple conspicuous veins. Seed cone with helically arranged, imbricated, and tightly interlocked bract-scale complexes on a slender central axis. Each bract-scale complex consisting of an inconspicuous bract partially fused to the stalk of an ovuliferous scale. Ovuliferous scale with five conspicuous spine-tipped lobes; three distal (always pointing away from the cone base), the other two proximal (always pointing toward the cone base). Bract scale complexes bearing three to five winged seeds. Leaves and bract-scale complexes with thin, delicate cuticles. Outlines of epidermal cells frequently arranged in two pairs, sometimes forming quartets. Stomatal complexes of both organs transversely oriented, paracytic and monocyclic. Outlines of the guard cells not sunken, flanked by the outlines of two lateral subsidiary cells.

Note: The family includes the seed cone genus *Krassilovia* and the leaf species *Podozamites harrisii* Shi, Herrera, Herendeen, Leslie, Ichinnorov, Takahashi et Crane.

## Discussion

### Relationships and evolution within the Krassilovia Clade

Cladistic analyses based on morphological and anatomical data allow the recognition of an unusual conifer group–the Krassilovia Clade–composed of the Triassic *Cycadocarpidium-Podozamites schenkii* plant, the Triassic–Jurassic *Swedenborgia-Podozamites schenkii* plant, and the Early Cretaceous *Krassilovia mongolica-Podozamites harrisii* plant. This new recognized group is unified by a combination of foliar features that include distichous strap-shaped leaves with transversely oriented paracytic stomata. Phylogenetic analyses suggest that the Krassilovia Clade is also characterized by thin cuticles with paracytic, non-sunken stomata. However, seed cones of Krassilovia Clade are morphologically diverse (see [49]). Seed cones of *C*. *erdmanni* and *S*. *cryptomerioides* [33, 49, 62, 67] (S2 Appendix) are elongated and lax, whereas those of *Krassilovia mongolica* are almost spherical, dense, and composed of imbricated and tightly interlocked bract-scale complexes (Fig 1). The five-lobed bract-scale complexes of *S*. *cryptomerioides* [33, 49] are somewhat similar to those of *Krassilovia*, but in *Swedenborgia* all the lobes are oriented distally (always pointing away from the cone base) while in *Krassilovia* only three of the five lobes are oriented distally, and the other two are proximal (always pointing toward the cone base: [49]; Fig 1). The bract-scale complexes of *C*. *erdmanni* are distinctive in having two lobes and a prominently developed and very long acuminate bract [62] (S2 Appendix). They are quite different from those of both *Krassilovia* and *Swedenborgia*.

### Implications for seed plant evolution

In a study of permineralized *Heidiphyllum* (= *Notophytum*; see [46]) leaves from the Triassic of Antarctica Axsmith et al. [75] suggested that *Aethophyllum*, *Borysthenia*, *Cycadocarpidium*, *Swedenborgia*, and *Telemachus* were part of a single group of “*transitional conifers*”, probably related to the Podocarpaceae. More recent phylogenetic approaches [48, 49] recognized a clade of Mesozoic conifers comprising *Aethophyllum*, *Krassilovia*, *Swedenborgia* and *Telemachus* (= *Parasciadopitys*; see [46]). The analyses presented here further refine this pattern with the recognition of the Krassilovia Clade as a discrete group of three closely related taxa related to the conifer crown group (Fig 5).

The most unusual trait of the Krassilovia Clade is the presence of transversely oriented paracytic stomata that resemble those of extant Gnetales, extinct Bennettitales (see [76]), and some angiosperms, rather than those of extant conifers (see also [63]). Paracytic stoma appear to have evolved more than once in plant evolution and vary in how they develop (mesogenous vs mesoperigenous, [76–78], however, the presence of such stomata in putative conifers and also in Gnetales is of interest given the close relationship suggested by recent phylogenetic analyses based on DNA data. For example, Rudall and Bateman [78] hypothesized that the closest living analogues to bennettitalean stomatal development occur in *Gnetum* and *Welwitschia* (with mesogene origin of the lateral subsidiary cells). The pinnae of many pinnately compound bennettitalean leaves (e.g., *Dictyozamites*, *Pterophyllum*) have paracytic stomata that are oriented transverse to the veins [76, 79–81], as in the leaves and bract-scale complexes of the *Krassilovia* plant (Fig 2). However, in *Welwitschia* stomata are oriented parallel to the veins, whereas in *Ephedra* and *Gnetum* they can occur in various orientations [76]. *Gnetum* is also unique among extant Gnetales in having a quartet epidermal prepatterning in which groups of four protodermal cells occur in a ‘squared’ arrangement ([76], *sensu* Rudall & Bateman [78]). The mature paired epidermal cells of *Gnetum*, (see [76]) are also strikingly similar to the mature paired epidermal cells of the leaves of *Podozamites harrisii* (Fig 2C) and the ovuliferous scales of *K*. *mongolica* (Fig 2F).

The putative relationship between the Krassilovia Clade and extant Gnetales deserves further scrutiny, but inclusion in our cladistic analysis of the *Dechellyia*-*Masculostrobus* plant (Fig 5B), a putative early gnetalean relative from the Triassic Chinle Formation, [66] (S4 Appendix), indicates that additional information on early Gnetales and the inclusion of other fossils (e.g., the gnetalean *Cearania heterophylla* from the Early Cretaceous Crato Formation in Brazil; [82]) in phylogenetic analyses may be relevant to understanding relationships among the Krassilovia Clade, Gnetales, and stem and crown conifers.

Fossils of *Dechellyia gormanii* consists of shoots with attached winged seeds and distichously arranged opposite and decussate leaves. The leaves are of two kinds: small, scale-like clasping leaves like those of many fossil and living conifers, and strap-shaped leaves, with two longitudinal veins (S4 Appendix). Unfortunately, cuticular details are not preserved. Pollen grains of *Equisetosporites chinleana*, which resemble those of extant *Ephedra* and *Welwitschia*, occur in the associated cones of *Masculostrobus clathratus* [66]. The *Dechellyia*-*Masculostrobus* plant presents an interesting combination of characters. While the pollen grains and the arrangement of the linear leaves are suggestive of Gnetales, the small clasping leaves and pollen cones are more suggestive of conifers. The winged seeds recall those of *Cycadocarpidium* and it is also interesting that the bract-scale complexes of *Cycadocarpidium swabi* [33, 67] are borne in an opposite and decussate arrangement. Further information on plants like *Dechellyia*, and plants that produced similar winged fossil seeds, such as *Fraxinopsis* [73], would be of great interest.

### Paleoecology of the *Krassilovia mongolica*–*Podozamites harrisii* plant

The vast number of disarticulated specimens recovered from the Tevshiin Govi lignite [49, 63] show that *P*. *harrisii* leaves were regularly shed and that the seed cones of *K*. *mongolica* disarticulated at maturity into dozens of individual cone scales from which the seeds were readily dispersed. The high M_a_ value of *P*. *harrisii* leaves (207.45 g/m^2^), and the scaling relationship between leaf mass and petiole width seen among living broadleaf gymnosperms [53–55, 83], suggests that *P*. *harrisii* leaves were likely evergreen and not annually deciduous. As in many extant broadleaved conifers the leaves probably abscised and fell at the end of their useful life [84]. In contrast, the lack of prominent xylem near the base of *Krassilovia* bract-scale complexes, and the relatively large lumens and thin walls of the xylems cells, suggest a more precise mode of dehiscence (Figs 6 and 7), analogous, for example, to the disarticulation of the cones and shedding of bract-scale complexes that occurs extant *Abies* and *Cedrus* [85]. A well-organized mode of shedding is also consistent with the very regular, distinct scars seen on dispersed *Krassilovia* seed cone axes (Fig 1D). During development of the cones the strongly imbricate spiny bract-scale complexes would have provided protection for the developing ovules/seeds, but seed release at maturity required programmed disarticulation [49].

The shed parts of the *Krassilovia* plant are a significant component of the Tevshiin Govi lignite, which shows no evidence of higher energy input and appears to have accumulated largely autochthonously. Growing alongside the *Krassilovia* plant were a variety of other presumed trees, including taxa related to extant Pinaceae (*Schizolepidopsis*, *Picea*, *Pityostrobus*, [65, 86]), Cupressaceae (*Elatides*, *Stutzeliastrobus*, *Pentakonos*, [64, 87], and the ginkgophyte *Umaltolepis* [70], as well as plants that may have been shrubs, such as the corystosperm *Umkomasia* [31, 32]. The absence of lycopods and ferns (as macrofossils, mesofossils, or megaspores), other than the epiphytic filmy fern *Hymenophyllum* [88], suggests there was little or no herbaceous vegetation. A miniscule moss (Herrera, F. personal observation), represented by only a few fragments in the Tevshiin Govi flora may also have been epiphytic.

Compared to modern vegetation the Tevshiin Govi swamp was unusual in its diversity and dominance of conifers and other groups of non-angiosperm seed plants. Gymnosperms rarely dominate swamp, bog, or a river floodplain environments today. Some living conifers, for example *Taxodium distichum* or *Picea mariana*, can form extensive swamp or bog forests [71, 89], but today the number of conifer species that co-occur in swamp habitats is very restricted, and conifers share these poorly drained habitats with angiosperms.

## Conclusions

*Krassilovia* and the Krassilovia Clade suggest the need to reevaluate current models of conifer evolution and reassess the significance of unusual morphological traits in living and fossil conifers. Current concepts of “conifers” as an evolutionary meaningful group may have been unduly influenced by their simple leaves. Furthermore, their other potential unifying feature, the compound ovulate shoot, is not diagnostic and occurs in other groups of living and fossil plants. In our cladistic analyses, the Krassilovia Clade appears to be close to the conifer crown group but it likely evolved from a paraphyletic and diverse assemblage of ancient conifers or conifer-like plants. Morphological differences among the seed cones of Krassilovia Clade, from elongated and lax in the *Cycadocarpidium-Podozamites* and *Swedenborgia-Podozamites* plants, to compact and tightly interlocked in the Early Cretaceous *Krassilovia-Podozamites* plant, highlight the diversity within the group. However, their conifer-like features, combined with their potential similarities to Gnetales, suggest new lines of investigation to further examine the close gnetalean-conifer relationship inferred from DNA data.

The analysis presented here provides only an initial assessment of the potential relationship of Gnetales, given the few alternative phylogenetic positions that were possible for the *Dechellyia-Masculostrobus* plant with such limited taxonomic sampling of other potentially relevant seed plants. Nevertheless, it is interesting that the *Dechellyia-Masculostrobus* plant is resolved close to the Krassilovia Clade even when most extant and fossil placeholders for extant families of conifers are excluded (S5 Appendix). Also, recognition of the Krassilovia Clade, which combines conifer-like cones with leaves that have transversely oriented paracytic stomata, highlights similarities to both conifers and Gnetales, as also do features of the *Dechellyia-Masculostrobus* plant. Ultimately, conifer monophyly may or may not be supported, but a more definitive understanding will require incorporating more fossil material into morphology-based phylogenetic analyses, not only putative conifers, but also other Gnetales, Bennettitales and Erdtmanithecales, as well as corystosperms and similar plants.

## Supporting information

S1 Appendix List of morphological characters. (DOCX)

S2 Appendix *Cycadocarpidium erdmanni* & *Podozamites schenkii*. (PDF)

S3 Appendix *Krassilovia* Tevshiin Govi. (PDF)

S4 Appendix *Dechellyia*-*Masculostrobus* plant. (PDF)

S5 Appendix Additional cladistic analyses. (PDF)

S6 Appendix *Krassilovia* Shine Khudag. (PDF)

S7 Appendix Neutron tomographic volume *Krassilovia*. (MOV)
