# Supplementary material for: Reconstructing Krassilovia mongolica supports recognition of a new and unusual group of Mesozoic conifers
Source: PLoS One. 2020 Jan 15;15(1):e0226779. doi: 10.1371/journal.pone.0226779 (PMC6961850; doi:10.1371/journal.pone.0226779)
Supplement: S1 Appendix — (DOCX) [file pone.0226779.s001.docx]

Rothwell et al. (2005) and Escapa et al. (2010) rooted their analysis on *Callistophyton*. We show the scoring for *Callistophyton* as part of our matrix but regard *Callistophyton* as strictly inapplicable for many of the characters scored in this analysis because of the very different fern-like leaves and ovulate and pollen structures compared to those of coniferophytes. Therefore, for current purposes we exclude *Callistophyton* from consideration and root the analysis on the two cordaitaleans taxa, *Cordaixylon* and *Mesoxylon*.

The selection of taxa, and much of their scoring is based on Rothwell et al. (2005). However, we add early fossil members of the Cupressaceae (*Elatides zhoui*) and Pinaceae (*Schizolepidopsis canicularis*) as placeholders for these extant groups. We also added extant taxa as further placeholders for extant families: *Cedrus deodara* for Pinaceae, *Cunninghamia lanceolata* for Cupressaceae and *Sciadopitys* *verticillata* for Sciadopityaceae. Finally, as a preliminary exploration of the potential relationship of Gnetales to conifers we assessed the position of a putative early gnetalean, the *Dechellyia gormanii*-*Masculostrobus clathratus* plant, among the other fossil and extant plants we considered.

Several characters are redefined and modified from the definitions of Rothwell et al. (2005) and Escapa et al. (2010), and various changes in scoring are noted in the descriptions of the character states. Scoring of *Swedenborgia* is as per Escapa et al. (2010), except where noted. Scoring of the *Telemachus* plant is based on Escapa et al. (2010) and Bomfleur et al. (2013). Scoring of *Krassilovia* is as per Herrera et al. (2015), except where noted and with the addition of information on the leaves. Scoring of *Cycadocarpidium* is based on the descriptions in Harris (1935) and Schweitzer & Kirchner (1996). Scoring of *Pseudovoltzia liebeana* is based on Clement-Westerhof (1987) and Schweitzer (1963). Scoring of *Manifera talaris* is based on Looy and Stevenson (2014).

The core analysis included 34 fossil and living taxa and 51 morphological characters (excluding character #52). We then conduct several simple experiments to explore the impact on potential relationships of including the *Dechellyia gormanii*-*Masculostrobus clathratus* plant and excluding the two extant placeholders and the two fossil representatives of Cupressaceae and Pinaceae (S5 Appendix).

We also experimented with the inclusion of two Cheirolepidiaceae, *Pararaucaria taquetrensis* (Escapa and Leslie, 2017) and *Frenelopsis ramosissima* (Axsmith and Jacobs, 2005), as well as the Permian conifer *Lebowskia grandifolia* (Looy, 2007) (S5 Appendix); this matrix included an additional morphological character (#52, see below).

**Additional References:**

Axsmith BJ, BF Jacobs 2005 The conifer *Frenelopsis ramosissima* (Cheirolepidiaceae) in the Lower Cretaceous of Texas: Systematic, biogeographical, and paleoecological implications. Int J Plant Sci 166: 327–337.

Escapa I, A Leslie 2017 A new Cheirolepidiaceae (Coniferales) from the Early Jurassic of Patagonia (Argentina): reconciling the records of impression and permineralized fossils. Am J Bot 104: 322–334.

Looy CV 2007 Extending the range of derived Late Paleozoic conifers: *Lebowskia* gen. nov. (Majonicaceae). Int J Plant Sci 168: 957–972.

Traverse A 2007 Paleopalynology (Vol. 28). Springer Science & Business Media.

**Deleted Characters from Rothwell et al. 2005.**

**3. Leaf dimorphism on the same branches**

Present (0); absent (1). Replaced by character 3.

**4. Leaves on penultimate branches different from leaves on ultimate**

Absent (0); Present (1). Replaced by character 3.

**6. Heteroblasty**

Absent or slight (0); prominent (1). Replaced by character 3.

**15. Leaf margin**

Entire (0); with uniseriate trichomes (1); toothed (at least biseriate immediately distal to base) (2). Replaced by character 17.

**18. Subsidiary cell positions**

Surrounding guard cells (0); lateral and often polar to guard cells (1). Replaced by character 13.

**19. Dicyclic stomata**

Absent (0); present (at least in part) (1). Replaced by character 13.

**21. Surficial trichomes**

Present (0); absent (1). Replaced by character 17.

**23. Pollen cones structure**

Simple shoots (0); compound shoots (1); no definition (2). Replaced by character 18.

**29. Adaxial pollen sac attachment**

Absent (0); present (1). Replaced by character 24.

**32. Protosacci of pollen**

Absent (0); present (1). Combined with character 33 from Rothwell et al. (2005) as character 25 in this analysis.

**33. Eusacci of pollen**

Absent (0); present (1). Combined with character 33 from Rothwell et al. (2005) as character 25 in this analysis.

**36. Number of ovules per UOU (ultimate ovule bearing unit).**

(0) >1; (1) 1. This character becomes uninformative when excluding *Callistophyton*. Note that *Cordaixylon* and *Ernestiodendron* should be corrected as state (1) in Rothwell et al. (2005).

**42. Marginally dentate bract**

Absent (0); present (1). Replaced by character 34

**44. Ovules produced**

From all sides of shoot (0); adaxially (i.e., facing apex of stem upon which axillary shoot is borne) and laterally (1); from lateral sides of shoot only (2); from adaxial side of shoot only (3). Replaced by character 39.

**Phenotypic characters (numbered in Nexus format)**

**1. Orthotropic branching of stem**

*[[Previously character 1 from Rothwell et al. (2005). Orthotropic growth indicates that the stem grows in a more or less vertical in orientation (as opposed to scrambling). Emporia cryptica and E. royalii were scored as (1), as Emporia spp. was treated by Rothwell et al. (2005). However, E. lockardii was scored as (?). Ernestiodendron was rescored from (1) to (?) due to lack of information. Genoites was rescored from (?) to (1) based on Cuneo (1985). Telemachus was scored as (?) by Escapa et al. (2010) and rescored as (1) based on Bomfleur et al. 2013. Dechellyia was scored as (1) based on Ash (1972).]]*

Absent (0); present (1).

**2. Ultimate branch arrangement**

*[[Previously character 2 from Rothwell et al. (2005). Irregular/three dimensional: refers to lateral branch systems in which the leafy shoots are arranged three dimensionally or irregularly with respect to the direction of growth of the main axis. Plagiotropic refers to lateral branch systems in which the leafy shoots are arranged in one plane more or less perpendicular to the direction of growth of the main axis and are often more or less pinnately arranged. All three species of Emporia are scored as (1), as Emporia spp. was treated by Rothwell et al. (2005). Telemachus was scored as (?) by Escapa et al. (2010), and rescored as (0) based on Bomfleur et al. 2013. Dechellyia was scored as (1) based on Ash (1972).]]*

Irregular/three dimensional (0); plagiotropic (pinnate) (1).

**3. Heterophylly**

*[[Previously characters 3 and 4 from Rothwell et al. (2005) now combined, stated differently and not attempting to discriminate different kinds of heterophylly. Heterophylly refers to pronounced differences in the shape of leaves, where two distinctive types of leaves are known (Hernandez-Castillo et al. 2009c). It may include position dependent, size dependent, and age dependent heterophylly (Hernandez-Castillo et al. 2009c). Dechellyia was scored as present (1) based on Ash (1972).]]*

Absent (0); present (1).

**4. Bud scales**

*[[Previously character 5 from Rothwell et al. (2005). Absent, refers to the development of a lateral shoot from a meristem not previously contained in a resting scaly bud. This type of branching, which results in the production of synleptic shoots, is common in plants with more or less continuous pattern of growth and is typical of many extinct Paleozoic conifers. Present, refers to the development of a lateral shoot from a meristem previously contained within a resting scaly bud. All three species of Emporia are scored (0), as Emporia spp. was treated by Rothwell et al. (2005). Dechellyia was scored as (?) based on Ash (1972).]]*

Absent (0); present (1).

**5. Vegetative leaf shape and venation**

*[[Previously character 7 from Rothwell et al. (2005). All three species of Emporia are scored as (0), as Emporia spp. was treated by Rothwell et al. (2005). Swedenborgia was scored as (?) by Escapa et al. (2010) and rescored as (1) based on Harris (1935) and Nosova et al. (2017). Krassilovia was scored as (?) by Herrera et al. (2015) and rescored as (1) based on the new evidence presented in this paper. An additional state –* broadly triangular, scale-like or cataphyll-like *(3) – is incorporated to accommodate the leaves of Sciadopitys. Dechellyia was scored as (1,3) based on Ash (1972)]]*

Relatively narrow with one or two veins (0); strap-shaped with more than two veins (1); pinnately compound with dichotomous venation (2); broadly triangular, scale-like or cataphyll-like (3).

**6. Shape of narrow leaves in lateral view**

*[[Previously character 8 from Rothwell et al. (2005). This character and its states were redefined to focus on major leaf shape characteristics. We apply this character only to narrow leaves with one or two veins (see character 5). Emporia lockardii was rescored as (1). Emporia cryptica was rescored as (1) based on Hernandez-Castillo et al. (2009b). E. royalii was rescored as (1) based on Hernandez-Castillo et al. (2009c). We regard those taxa that have strap-shaped leaves with more than two veins, or pinnately compound leaves with dichotomous venation (see character 5), as inapplicable for this character*. *Dechellyia was scored as (0) based on Ash (1972).]]*

Straight, linear (0); short, slightly curved, S-shaped, falcate (1).

**7. Leaves narrowing toward base**

*[[Previously character 9 from Rothwell et al. (2005). All three species of Emporia are scored as (0), as Emporia spp. was treated by Rothwell et al. (2005). Aethophyllum was rescored from (0) to (1) based on Grauvogel-Stamm (1978) and Rothwell et al. (2000). The Vojnovskyean plant was rescored from (1) to (0) based on Rothwell et al. (1996). Krassilovia was scored as (?) by Herrera et al. (2015), and rescored as (1) based on the new evidence presented in this paper. Dechellyia was scored as (1) based on the linear leaves illustrated by Ash (1972).]]*

Absent (0); present (1).

**8. Cross-section of narrow leaves with one or two veins**

*[[Previously character 10 from Rothwell et al. (2005). We assess this character based on the cross-sections presented in the publications, although we caution that the published cross sections vary in their position on the leaf. All three Emporia species were scored as (0), as Emporia spp. was treated by Rothwell et al. (2005). Thucydia was rescored from (1) to (0,3) based on Hernandez-Castillo et al. (2001), resulting in no taxa being scored as (1). Concholepis was rescored from (2) to (?) based on Meyen (1997). We score those taxa that have strap-shaped leaves with more than two veins or pinnately compound leaves with dichotomous venation (see character 5) as inapplicable for this character. Dechellyia was scored as (?) based on Ash (1972).]]*

Rhomboid and transversely elongated (0); ellipsoidal with larger, convex adaxial face (1); thin and flattened (2); vertically elongated, diamond-shaped (3).

**9. Leaf apex**

*[[Previously character 11 from Rothwell et al. (2005). Character state (2, mucronate) of Rothwell et al. (2005) does not occur in any of the taxa included here, or in any of the taxa included in the original analysis. All three Emporia species were scored as (0), as Emporia spp. was treated by Rothwell et al. (2005). Aethophyllum was rescored from (1) to (0) based on Grauvogel-Stamm (1978) and Rothwell et al. (2000). The Vojnovskyean plant was rescored from (1) to (0) based on Rothwell et al. (1996). Krassilovia was scored as (?) by Herrera et al. (2015) and rescored as (1) based on the new evidence presented in this paper. We regard Callistophyton as inapplicable for this feature. Dechellyia was scored as (0) based on Ash (1972).]]*

Acute or pointed (0); rounded (1).

**10. Leaf cushions or consistently shaped leaf scars**

*[[Previously character 12 from Rothwell et al. 2005. All three Emporia species were scored as (0), as Emporia spp. was treated by Rothwell et al. (2005). Telemachus was scored as (?) by Escapa et al. (2010), and rescored as (1) based on Bomfleur et al. 2013. Krassilovia was scored as (?) by Herrera et al. (2015), and rescored as (1) based on the new evidence presented in this paper. Dechellyia was scored as (?) based on Ash (1972).]]*

Absent (0); present (1).

**11. Forked leaves on penultimate** **branches**

*[[Previously character 13 from Rothwell et al. (2005). This character and its states were redefined to focus on forking of the leaf rather than leaf margin characteristics. We also correct the definition to specify "penultimate" instead of "ultimate" branches (to differentiate this character from character 12 [below], character 14 of Rothwell et al. 2005). Emporia cryptica (Hernandez-Castillo et al. 2009b) was rescored as (0), rather than (1) as Emporia spp. was treated by Rothwell et al. (2005). Emporia lockardii and Emporia royalii were both rescored as (0,1) based on Hernandez-Castillo et al. (2009a, c). Dicranophyllum was rescored from (2) to (1) as a result of our character redefinition. Krassilovia was scored as (?) by Herrera et al. (2015) and rescored as (0) based on the new evidence presented in this paper. We regard Callistophyton as inapplicable for this feature. Dechellyia was scored as (0) based on Ash (1972).]]*

Not forked (0); forked (1).

**12. Forked leaves on ultimate branches**

*[[Previously character 14 from Rothwell et al. (2005). This character was redefined to focus on forking of the leaf rather than leaf margin characteristics. Emporia cryptica and Emporia royalii were scored as (0), as Emporia spp. was treated by Rothwell et al. (2005). Emporia lockardii was rescored from (0) to (0,1) based on Hernandez-Castillo et al. (2009a). Dicranophyllum was rescored from (2) to (1) as a result of our character redefinition. Krassilovia was scored as (?) by Herrera et al. (2015) and rescored as (0) based on the new evidence presented in this paper. We regard Callistophyton as inapplicable for this feature. Dechellyia was scored as (0) based on Ash (1972).]]*

Not forked (0); forked (1).

**13. Stomatal subsidiary cells**

*[[This new character reformulates characters 18 and 19 from Rothwell et al. (2005). All taxa were reviewed and rescored according to the new states.*

Unspecialized, in a simple ring (0); specialized, in two distinct rings (dicyclic - at least in part) (1); specialized, two lateral, two polar (*Cordaites*-type) (2); specialized, two lateral (parallel to the guard cells: paracytic) (3).

**14. Arrangement of stomata on adaxial cuticle**

*[[Previously character 16 from Rothwell et al. (2005). Careful assessment of the relevant publications resulted in significant rescoring of this character. Cordaixylon was rescored from (2) to (0) based on Rothwell & Warner (1984) and Mesoxylon was rescored from (2) to (3) based on Trivett & Rothwell (1985). All three Emporia species were scored as (1), as Emporia spp. was treated by Rothwell et al. (2005). Majonica was rescored from (3) to (3,4) based on Clement-Westerhof (1987). Ortiseia spp. was rescored from (3) to (0,1,3) based on Clement-Westerhof (1984). The Vojnovskyean plant was rescored from (0) to (3) based on Rothwell et al. (1996). Telemachus was scored as (4) by Escapa et al. (2010), and rescored as (0) based on Bomfleur et al. (2013). Krassilovia was scored as (?) by Herrera et al. (2015), and rescored as (0) based on the new evidence presented in this paper. Dechellyia was scored as (?) based on Ash (1972).]]*

Absent (0); two bands (1); multiple bands (2); uniseriate rows (3); scattered (4).

**15. Arrangement of stomata on abaxial cuticle**

*[[Previously character 17 from Rothwell et al. (2005). Careful assessment of the relevant publications resulted in significant rescoring of this character. Cordaixylon was rescored from (0) to (2) based on Rothwell & Warner (1984) and Mesoxylon was rescored from (0) to (3) based on Trivett & Rothwell (1985). Emporia cryptica was rescored from (4) to (1,3) based on Hernandez-Castillo et al. (2009b). Emporia lockardii was rescored as (1) based on Hernandez-Castillo et al. (2009a). Emporia royalii was rescored as (3) based on Hernandez-Castillo et al. (2009c). Majonica was rescored from (3) to (3,4) based on Clement-Westerhof (1987). Kungarodendron was rescored from (0) to (4) based on Meyen (1997). Ortiseia spp. was rescored from (3) to (1,3) based on Clement-Westerhof (1984). The Vojnovskyean plant was rescored from (2) to (0) based on Rothwell et al. (1996). Swedenborgia was rescored from (4) to (2) based on Nosova et al. (2017). Krassilovia was scored as (?) by Herrera et al. (2015), and rescored as (2) based on Shi et al. (2018) and the new evidence presented in this paper. Dechellyia was scored as (?) based on Ash (1972).]]*

Absent (0); two bands (1); multiple bands (2); uniseriate rows (3); scattered (4).

**16. Subsidiary cells with prominent papillae**

*[[Previously character 20 from Rothwell et al. (2005). Careful assessment of the relevant publications resulted in significant rescoring of this character. The character state scores for this character were reversed by Rothwell et al. (2005) and have been corrected in the new matrix. All three Emporia species are alike in having papillae (0). Swedenborgia was rescored from (?) to (1) based on Harris (1935) and Nosova et al. (2017). Telemachus was scored as (?) by Escapa et al. (2010) and rescored as (1) based on Bomfleur et al. (2013). Krassilovia was scored as (?) by Herrera et al. (2015) and rescored as (1) based on Shi et al. (2018) and the new evidence presented in this paper. Dechellyia was scored as (?) based on Ash (1972).]]*

Present (0); absent (1).

**17. Trichomes**

*[[This new character replaces characters 15 and 21 from Rothwell et al. (2005). Note that trichomes on the bracts are scored separately (see character 35). The scoring adopted here captures the different distributions of trichomes on different parts of the plant body. Dechellyia was scored as (?) based on Ash (1972).]]*

Absent (0); present along leaf margin (1); present on leaf surfaces (abaxial or adaxial) (2); present on reproductive surfaces (3); present on stems (4).

**18. Pollen cone structure**

*[[This new character reformulates character 23 from Rothwell et al. (2005). No definition for state (2) was given by Rothwell et al. (2005), but the condition in Cordaixylon and Mesoxylon was scored as (2), the same as in Thucydia. Given the structural differences, we think it is unlikely that the pollen cones of these three taxa are homologous. We therefore distinguish the compound condition in Cordaixylon and Mesoxylon (pollen cones with fertile axillary buds in four ranks; character state (1)) from that in Thucydia (pollen cones are compound shoots comprising helically arranged dwarf shoots in the axils of bracts on a main axis. Polliniferous dwarf shoots produce sterile scales and sporophylls with a terminal pollen sac; character state (2)). All taxa were reviewed and rescored according to the new states. We regard Callistophyton as inapplicable for this feature. Dechellyia was scored as (0) based on Ash (1972).]]*

A simple shoot with helical microsporophylls (0); compound shoot with fertile axillary buds in four ranks (1); compound shoot of the *Thucydia*-type (*helically arranged dwarf shoots in the axils of bracts on a main axis and sporophylls with a terminal pollen sac*) (2).

**19. Position of pollen cone**

*[[Previously character 22 from Rothwell et al. (2005). Rothwell et al. (2005) used three states, and several taxa were scored as (2), but state "2" was not defined. All taxa were reviewed and rescored according to the two states we recognize. We regard Callistophyton as inapplicable for this feature. Dechellyia was scored as (1) based on Ash (1972).]]*

Lateral, in the axil of leaf (0); terminal (1).

**20. Differentiation of microsporophyll into shank and distal lamina**

*[[Previously character 24 from Rothwell et al. (2005). We recognize two states. Aethophyllum and Barthelia were rescored from (1) to (0) based on Grauvogel-Stamm (1978) and Rothwell & Mapes (2001) respectively. Cordaixylon and Mesoxylon were rescored from (0) to (1) based on Rothwell (1993) and Trivett & Rothwell (1985) respectively. All three species of Emporia were rescored from (1), as Emporia spp. was treated by Rothwell et al. (2005) to (0) based on Hernandez-Castillo et al. (2009a,b,c). Kungurudendron and Ortiseia were rescored from (?) to (0) based on Meyen (1997) and Clement-Westerhof (1984) respectively. Otovicia was rescored from (1) to (?) based on Kerp et al. (1990). Thucydia was rescored from (0) to (1) based on Hernandez-Castillo et al. (2001).*

*Telemachus was rescored from (?) (Escapa et al. 2010) to (0) based on Bomfleur et al. (2011). We regard Callistophyton as inapplicable for this feature. Dechellyia was scored as (0) based on Ash (1972).]]*

Present (0); absent (1).

**21. Symmetry of the microsporophyll**

*[[Previously character 25 from Rothwell et al. (2005), which we follow in recognizing two states: ‘linear’ and ‘distal lamina bilateral’. Rothwell et al. (2005) defined two additional states: "with radial distal lamina, peltate (2)" and "with entire sporophyll laminar (3)", but these states were not scored for any of the taxa in their matrix. We regard Callistophyton as inapplicable for this feature. Dechellyia was scored as (1) based on Ash (1972).]]*

Linear (0); distal lamina bilateral (1).

**22. Basal keel on the microsporophyll distal lamina**

*[[Previously character 26 from Rothwell et al. (2005). In this matrix we follow Rothwell et al. (2005) in scoring two states. Taxa scored (1) for character 20 are also regarded as inapplicable. All three Emporia species were scored as (1), as Emporia spp. was treated by Rothwell et al. (2005). Barthelia was rescored as (?) based on Rothwell & Mapes (2001). Kungurudendron was rescored as (?) based on Meyen (1997). Telemachus was scored as (?) by Escapa et al. (2010) and rescored as (0) based on Bomfleur et al. (2011, 2013). We regard Callistophyton as inapplicable for this feature. Dechellyia was scored as (0) based on Ash (1972).]]*

Absent (0); present (1).

**23. Microsporophyll apex forked**

*[[Previously character 27 from Rothwell et al. (2005). Telemachus was scored as (?) by Escapa et al. (2010), and rescored as (0) based on Bomfleur et al. (2011, 2013). Among the taxa we consider the microsporophyll apex is forked only in Barthelia. We regard Callistophyton as inapplicable for this feature. In addition, because the apices of the microsporophylls in Cordaixylon, Mesoxylon and Thucydia bear one or more pollen sacs (character 24) we also regard them as inapplicable. Dechellyia was scored as (0) based on Ash (1972).]]*

Microsporophyll apex not forked (0); microsporophyll apex forked (1).

**24. Pollen sac attachment**

*[[Previously characters 28 and 29 from Rothwell et al. (2005). In this matrix, we recognize four states modifying the states used by Rothwell et al. (2005) to recognize the possibility that the pollen sac attachment in Cordaixylon and Mesoxylon is not homologous to that Thucydia as it was presented in Rothwell et al. (2005). We also eliminate state (3) of character 28 of Rothwell et al. (2005) “adaxial surface of lamina” since no taxa were scored (3). All taxa were revised and rescored according to the new states. Dechellyia was scored as (3) based on Ash (1972).]]*

On abaxial leaf lamina (0); singly at the sporophyll tip with one terminal erect pollen sac (1); in a group at tip of sporophyll (2); sporophyll stalk or distal lamina with sacs on abaxial side (3).

**25. Presence/absence and type of sacci in prepollen/pollen**

*[[This new character replaces characters 32 and 33 from Rothwell et al. (2005). This scoring differentiates the different forms of sacci and is consistent with that of Rothwell et al. (2005) except for Ortiseia spp., which is rescored as protosaccate (1), and Otovicia hypnoides, which is rescored as eusaccate (0). Protosaccate refers to an extinct form of saccate gymnosperm pollen in which the sacci have extensive ektexinous webbing in the sacci, in contrast to eusaccate pollen, such as modern Pinus,*

*in which the sacci are nearly hollow (Traverse, 2007). Dechellyia was scored as (2) based on (Ash 1972).]]*

Eusacci (0); protosacci (1); lacking sacci (2).

**26. Prepollen/pollen with proximal** **suture**

*[[Previously character 30 from Rothwell et al. (2005). All three Emporia species are alike in having a proximal suture (0) as Emporia spp. was treated by Rothwell et al. (2005). Telemachus was scored as (?) by Escapa et al. (2010), and rescored as (1) based on Bomfleur et al. (2011, 2013). Dechellyia was scored as (1) based on (Ash 1972).]]*

Present (0); absent (1).

**27. Prepollen/pollen with distal aperture**

*[[Previously character 31 from Rothwell et al. (2005). All three Emporia species are alike in not having a distal aperture (0) as Emporia spp. was treated by Rothwell et al. (2005). Telemachus was scored as (?) by Escapa et al. (2010), and rescored as (1) based on Bomfleur et al. (2011, 2013). Dechellyia was scored as (1) based on Ash (1972).]]*

Absent (0); present (1).

**28. Ultimate ovule bearing unit**

*[[Previously character 34 from Rothwell et al. (2005). Character state (1) assumes that the ovules in Ferugliocladus and Genoites are attached to a small stalk that arises directly from the axis (stem) of a leafy shoot. This state (1) also assumes that the stalked ovules represent an extremely reduced dwarf shoot in which all of the scale leaves have been lost (Archangelsky & Cúneo, 1987).* *All three Emporia species are alike in having the ultimate ovule bearing unit borne on a leaf (0), as Emporia spp. was treated by Rothwell et al. (2005). Dechellyia was scored as (0) based on Ash (1972).]]*

Leaf (0); stem (1).

**29. Position of ultimate ovule bearing unit (UOU)**

*[[Previously character 35 from Rothwell et al. (2005). Rothwell et al. (2005) noted that "This [apical] is opposed to being lateral or axillary and is scored as present even if the apex grows through to be vegetative later." All three Emporia species are alike in having the ultimate ovule bearing unit borne on a leaf (0), as Emporia spp. was treated by Rothwell et al. (2005). We also regard Callistophyton as inapplicable for this feature. Dechellyia was scored as (1) based on Ash (1972).]]*

Lateral or axillary (0); terminal (1).

**30. Formation of fertile aggregations**

*[[Previously character 37 from Rothwell et al. (2005). In this matrix we have restated the character name and states, but the essence of the character definition and scoring remains the same. Taxa scored (0) indeterminate have fertile zones rather than discrete cones. Taxa scored as (1) determinate have discrete cones in the formation of which the apical meristem is generally used up. All three Emporia species are alike in having determinate cones (1), as Emporia spp. was treated by Rothwell et al. (2005). Concholepis was rescored from (?) to (0) based on Meyen (1997). The Vojnovskyean plant was rescored from (1) to (0) based on Taylor et al. 2009. We regard Callistophyton as inapplicable for this feature. Dechellyia was scored as (1) based on Ash (1972).]]*

Indeterminate (0); determinate (1).

**31. Leaf/bract subtending ovule or ovulate cone modified compared to vegetative leaf**

*[[Previously character 38 from Rothwell et al. (2005). All three Emporia species are alike in having a bract that is modified compared to the vegetative leaf (1), as Emporia spp. was treated by Rothwell et al. (2005). We regard Callistophyton as inapplicable for this feature. Dechellyia was scored as (1) based on Ash (1972).]]*

Not modified (0); modified (1).

**32. Fusion of leaf/bract subtending ovule or ovulate cone**

*[[Previously character 39 from Rothwell et al. (2005). Emporia cryptica and Emporia royalii were rescored from (0), as Emporia spp. was treated by Rothwell et al. (2005), to (1). Emporia lockardii was scored as (0), as Emporia spp. was treated by Rothwell et al. (2005). We regard Callistophyton as inapplicable for this feature. Dechellyia was scored as (?) based on Ash (1972).]]*

Free (0); fused only at base (1); fused for two-thirds or more the length of the bract (2).

**33. Apex** **of bract subtending ovule or ovulate cone**

*[[Previously character 40 from Rothwell et al. (2005). The character state scores for this character were reversed by Rothwell et al. (2005) and have been corrected in the matrix. All three Emporia species are alike in having a forked bract (1), as Emporia spp. was treated by Rothwell et al. (2005). Dicranophyllum was rescored from (0) to (1) based on Barthel (1977). Ernestiodendron was rescored from (0) to (1) based on Florin (1938-1945). The Vojnovskyean plant was rescored from (?) to (0) based on Rothwell & Mapes (1996). We regard Callistophyton as inapplicable for this feature. Dechellyia was scored as (0) based on Ash (1972).]]*

Entire (0); forked (1).

**34. Marginal trichomes on bract**

*[[Previously character 41 from Rothwell et al. (2005). This character also incorporates character 42 (marginally dentate bract) from Rothwell et al. (2005) since the dentations differ little from marginal trichomes. All three Emporia species are alike in having marginal trichomes on the bract (1), as Emporia spp. was treated by Rothwell et al. (2005). Aethophyllum was rescored from (?) to (0) based on Grauvogel-Stamm (1978). Concholepis was rescored from (?) to (0) based on Meyen (1997). Kungurodendron was rescored from (?) to (1) based on Meyen (1997). Thucydia was rescored from (0) to (1) based on Hernandez-Castillo et al. (2001). Telemachus was scored as (?) by Escapa et al. (2010) and rescored as (0) based on Bomfleur et al. (2013). We regard Callistophyton as inapplicable for this feature. Dechellyia was scored as (?) based on Ash (1972).]]*

Absent (0); present (1).

**35. Symmetry of axillary shoot/ovuliferous scale**

*[[Previously character 43 from Rothwell et al. (2005). All three Emporia species are alike in having a flattened (bilateral) axillary shoot (1), as Emporia spp. was treated by Rothwell et al. (2005). We regard Callistophyton as inapplicable for this feature. Dechellyia was scored as (1) based on Ash (1972).]]*

Radial (0); flattened (bilateral) (1).

**36. Ovule orientation**

*[[Previously character 45 from Rothwell et al. (2005). All three Emporia species are alike in having inverted ovules (1), as Emporia spp. was treated by Rothwell et al. (2005). Telemachus was scored as (?) by Escapa et al. (2010) and rescored as (1) based on Bomfleur et al. (2013). Dechellyia was scored as (0) based on Ash (1972).]]*

Erect (0); inverted (1).

**37. Ovules on distinct sporophylls**

*[[Previously character 46 from Rothwell et al. (2005). This character definition and its states are slightly redefined from Rothwell et al. (2005), but the essence of the character remains the same. This character refers to the presence of a distinct, visible, leaflike organ (albeit sometimes slightly modified) that bears the ovule(s). This is opposed to having a highly reduced and modified ovule-bearing structure, such as the ovuliferous scale in more derived conifers. All three Emporia species are alike in having ovules borne on distinct sporophylls (0), as Emporia spp. was treated by Rothwell et al. (2005). Dechellyia was scored as (1) based on Ash (1972).]]*

On distinct sporophylls (0); not on distinct sporophylls (1).

**38. Ovules borne on more-or-less fused structure**

*[[Previously character 47 from Rothwell et al. (2005). As noted by Rothwell et al. (2005) the “fused structure may represent take the form of a single structure, as is the case in Krassilovia, or it may more obviously take the form of several sporophylls and vegetative scales” (i.e., "fertile scales" of some authors; Mapes & Rothwell 1991) or an ovuliferous scale.”. Ovules on Ferugliocladus and Genoites are not considered to be borne on a more-or-less fused structure. All three Emporia species are alike in having ovules not borne on borne on a more-or-less fused structure (0), as Emporia spp. was treated by Rothwell et al. (2005). Dechellyia was scored as (?) based on Ash (1972).]]*

Not on a more-or-less fused structure (0); on a more-or-less fused structure (1).

**39. Ovule position on the fertile shoot**

*[[This new character is very similar to, but reformulates, character 44 from Rothwell et al. (2005). All taxa were reviewed and rescored according to the new states. We also introduce state 3, terminal on the shoot, (equivalent to the [undefined] state 4 of Rothwell et al. 2005), to accommodate the condition in Ferugliocladus and Genoites. We regard Callistophyton as inapplicable for this feature. Dechellyia was scored as (1) based on Ash (1972).]]*

On all sides of shoot (0); on the adaxial side of the shoot (1); lateral on the shoot (2); terminal on the shoot (3).

**40. Position of ovules on sporophyll/fertile scale/ovuliferous scale**

*[[Previously character 48 from Rothwell et al. (2005). All three Emporia species are alike in having apical/marginal ovules not borne on a more-or-less fused structure (0), as Emporia spp. was treated by Rothwell et al. (2005). Majonica was rescored from (3) to (2,3) based on Clement-Westerhof (1987). Swedenborgia and Telemachus were scored as (?) by Escapa et al. (2010) and rescored as (2) based on Harris (1935, 1937) and Bomfleur et al. (2013) respectively. We regard Callistophyton as inapplicable for this feature. Dechellyia was scored as (3) based on Ash (1972).]]*

Apical/marginal (0); surficial, near tip (1); surficial, mid-region (2); surficial, near base (3).

**41. Number of potentially fertile ovules per ovuliferous shoot/ovuliferous scale***[[Previously character 49 from Rothwell et al. (2005). State "(1) numerous" in Rothwell et al. (2005) was corrected to (0). Emporia cryptica and Emporia royalii are rescored from (1), as Emporia spp. was treated by Rothwell et al. (2005), to (3) based on Hernandez-Castillo (2009b, c). Emporia lockardii was rescored from (1), as Emporia spp. was treated by Rothwell et al. (2005), to (2) based on Hernandez-Castillo (2009a). Telemachus was scored as (?) by Escapa et al. (2010) and rescored as (1) based on Bomfleur et al. (2013). We regard Callistophyton as inapplicable for this feature. Dechellyia was scored as (4) based on Ash (1972).]]*

Numerous (0); variable, less than eight (1); three (2); two (3); one (4).

**42. Number of sterile vegetative scales on ovuliferous shoot**

*[[Previously character 50 from Rothwell et al. (2005). The character name has been slightly modified. None of the taxa in Rothwell et al. (2005) were scored as “(2)”. All three Emporia species are alike in having numerous sterile vegetative on the ovuliferous scale (0), as Emporia spp. was treated by Rothwell et al. (2005). Hanskerpia was rescored from (0) to (1) based on Rothwell et al. (2005) and Hernandez-Castillo et al. (2009c). Voltzia was rescored from (0) to (2) based on Schweitzer (1996). Telemachus was scored as (?) by Escapa et al. (2010) and rescored as (3) based on Bomfleur et al. (2013). We regard Callistophyton as inapplicable for this feature. Dechellyia was scored as (3) based on Ash (1972).]]*

Numerous, more than 20 (0); several, 6-20 (1); few, 1-5 (2); absent (3).

**43. Intergrading vegetative and fertile scales**

*[[Previously character 51 from Rothwell et al. (2005). All three Emporia species are alike in having intergrading vegetative and fertile scales (1), as Emporia spp. was treated by Rothwell et al. (2005). Barthelia, Concholepis, Hanskerpia, Ortiseia spp., Otovicia and Timanostrobus were rescored from (0) to (1) based on Rothwell & Mapes (2001), Meyen (1997), Rothwell et al. (2005), Clement-Westerhof (1984), Kerp et al. (1990) and Meyen (1997) respectively. We regard Callistophyton as inapplicable for this feature. Dechellyia was scored as (0) based on Ash (1972).]]*

Absent (0); present (1).

**44. Vegetative scales on adaxial surface of ovuliferous shoot**

*[[Previously character 52 from Rothwell et al. (2005). All three Emporia species are alike in having vegetative scales on the adaxial surface of the ovuliferous shoot (0), as Emporia spp. was treated by Rothwell et al. (2005). Dolomitia and Majonica were rescored from (1) to (0) based on Clement-Westerhof (1987). Ferugliocladus spp. was rescored from (0) to (1) based on Archangelsky & Cuneo (1987). Swedenborgia and Telemachus were scored as (0) by Escapa et al. (2010) and rescored as (1) based on Harris (1935, 1937) and Bomfleur et al. (2013) respectively. We regard Callistophyton as inapplicable for this feature. Dechellyia was scored as (1) based on Ash (1972).]]*

Present (0); absent (1).

**45. Vegetative scales on abaxial surface of ovuliferous shoot**

*[[Previously character 53 from Rothwell et al. (2005). All three Emporia species are alike in having vegetative scales on the abaxial surface of the ovuliferous shoot (0), as Emporia spp. was treated by Rothwell et al. (2005). Ferugliocladus spp. was rescored from (0) to (1) based on Archangelsky & Cuneo (1987). Swedenborgia and Telemachus were scored as (0) by Escapa et al. (2010) and rescored as (1) based on Harris (1935, 1937) and Bomfleur et al. (2013) respectively. We regard Callistophyton as inapplicable for this feature. Dechellyia was scored as (1) based on Ash (1972).]]*

Present (0); absent (1).

**46. Sporophylls aggregated on shoot apex to form a terminal cone**

*[[Previously character 54 from Rothwell et al. (2005). All three Emporia species are alike in having sporophylls aggregated on the shoot apex (0), as Emporia spp. was treated by Rothwell et al. (2005).*

*Barthelia was rescored from (?) to (1) based on Rothwell & Mapes (2001). Barthellia was rescored from (?) to (1), and Ernestiodendron was rescored from (1) to (0), based on based on Hernandez-Castillo et al. (2009c). Ferugliocladus and Genoites are scored as (1) noting that “the compound ovuliferous cones of these genera are simpler that those of other Paleozoic coniferophytes because the axillary ovuliferous dwarf shoots consist of only an axis with a terminal ovule” (Rothwell & Mapes 2001). We regard Callistophyton as inapplicable for this feature. Dechellyia was scored as (0) based on Ash (1972).]]*

Present (0); absent (1).

**47. Distinct stalk on ovuliferous shoot/ovuliferous scale**

*[[This new character recognizes a distinctive stalk located at the base of the ovuliferous shoot/ovuliferous scale. We interpret the condition in Genoites and Ferugliocladus as also having a stalk. We regard Callistophyton as inapplicable for this feature. Dechellyia was scored as (0) based on Ash (1972).]]*

Absent (0); present (1).

**48. Ovule apex**

*[[Previously character 55 from Rothwell et al. (2005) with only slight modification of the character name and states. All three Emporia species are alike in having an unmodified ovule apex (0), as Emporia spp. was treated by Rothwell et al. (2005). Swedenborgia and Telemachus were scored as (?) by Escapa et al. (2010) and rescored as (0) based on Harris (1935, 1937) and Bomfleur et al. (2013) respectively. Dechellyia was scored as (0) based on Ash (1972).]]*

Not modified (0); with two apical lobes or projections (1).

**49. Chalazal extensions on ovules (horns, lobes, wings etc.)**

*[[Previously character 56 from Rothwell et al. (2005). Emporia cryptica and Emporia lockardii were rescored from (1), as Emporia spp. was treated by Rothwell et al. (2005), to (3) based on Hernandez-Castillo (2009b, c). Emporia royalii was rescored from (1), as Emporia spp. was treated by Rothwell et al. (2005), to (0) based on Hernandez-Castillo et al. (2009c). Majonica was rescored as (1,3) based on Clement-Westerhof (1987). Swedenborgia was scored as (?) by Escapa et al. (2010), and rescored as (1,3) based on Harris (1935, 1937). Telemachus were scored as (?) by Escapa et al. (2010), and rescored as (0) based on Bomfleur et al. (2013). Dechellyia was scored as (0) based on Ash (1972).]]*

Absent (0); two extensions (1); three extensions (2); one, wing-like extension (3); absent, but seed wing derived from the ovuliferous scale (4).

**50. Coarse exterior trichomes on ovules**

*[[Previously character 57 from Rothwell et al. (2005). All three Emporia species are alike in having coarse exterior trichomes (1), as Emporia spp. was treated by Rothwell et al. (2005). Barthelia was rescored from (?) to (0) based on Rothwell & Mapes (2001). Swedenborgia was scored as (?) by Escapa et al. (2010), and rescored as (0) based on Harris (1935, 1937). Telemachus was scored as (?) by Escapa et al. (2010) and rescored as (0) based on Bomfleur et al. (2013). Dechellyia was scored as (?) based on Ash (1972).]]*

Absent (0); present (1).

**51. Phyllotaxy**

*[[This new character recognizes the placement of adjacent leaves on the nodes of the axis. Helical (spiral) refers to leaves arranged in a spiral along the axis; distichous (subopposite to opposite) refers to leaves (arranged in one plane in two ranks on opposite sides of the axis); decussate (opposite) refers to leaf pairs inserted at ~90∘ to those above and below). Dechellyia was scored as (3) based on Ash (1972).]]*

Helical (spiral) (0); distichous (subopposite to opposite) (1); decussate (opposite) (2).

**52. Ovuliferous scale with a “pocket-forming tissue"**

*[[This new character recognizes the presence of a flap of “pocket-forming tissue” (as defined by Escapa et al. 2012) on the adaxial side of the ovuliferous scale that loosely covers the seeds/ovules.]]*

Absent (0); Present (1).
