# Supplementary material for: Reconstructing Krassilovia mongolica supports recognition of a new and unusual group of Mesozoic conifers
Source: PLoS One. 2020 Jan 15;15(1):e0226779. doi: 10.1371/journal.pone.0226779 (PMC6961850; doi:10.1371/journal.pone.0226779)
Supplement: S2 Appendix — (PDF) [file pone.0226779.s002.pdf]

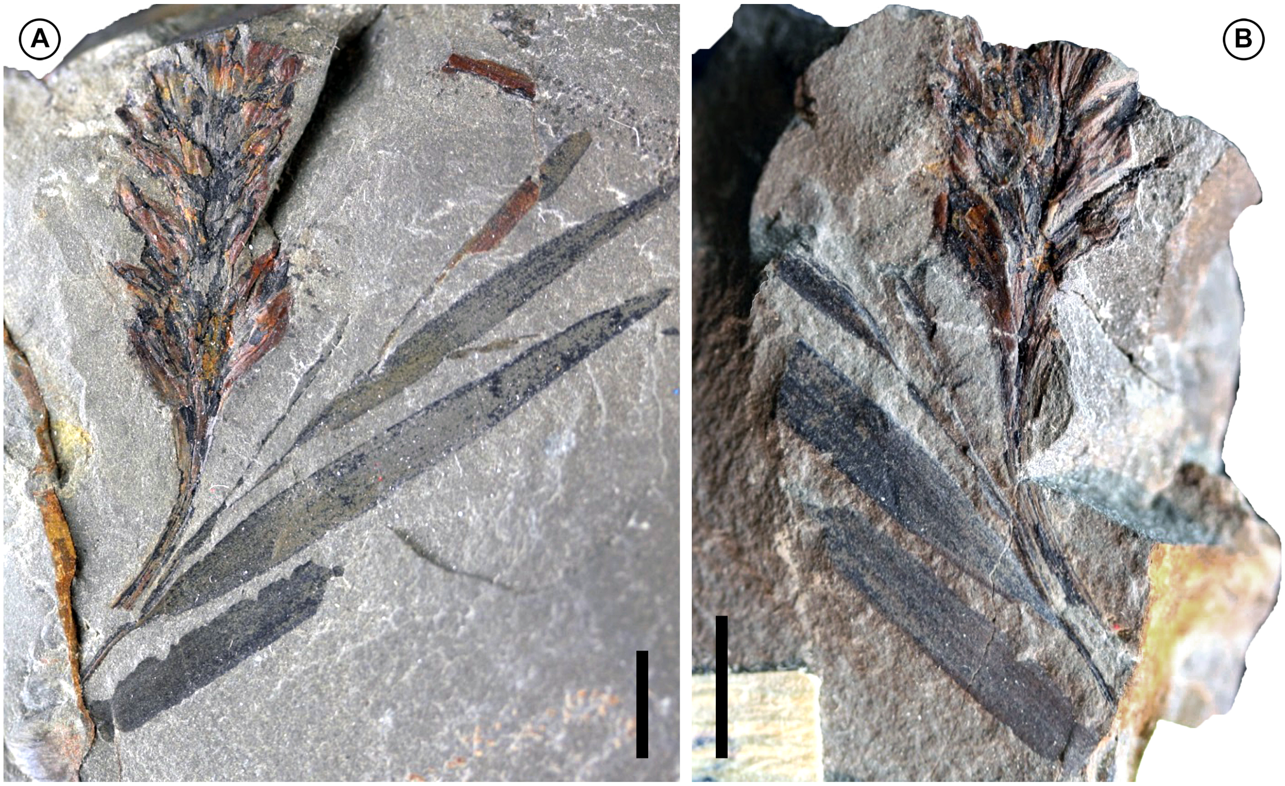

Cycadocarpidium erdmanni **(ovuliferous cone)-*****Podozamites schenkii*** **(leaf) based on Schweitzer and Kirchner (1996). (A): JE-Sch1286; (B): JE-Sch1287. Scale bars = 5 mm.**
