# Supplementary material for: Reconstructing Krassilovia mongolica supports recognition of a new and unusual group of Mesozoic conifers
Source: PLoS One. 2020 Jan 15;15(1):e0226779. doi: 10.1371/journal.pone.0226779 (PMC6961850; doi:10.1371/journal.pone.0226779)
Supplement: S3 Appendix — (PDF) [file pone.0226779.s003.pdf]

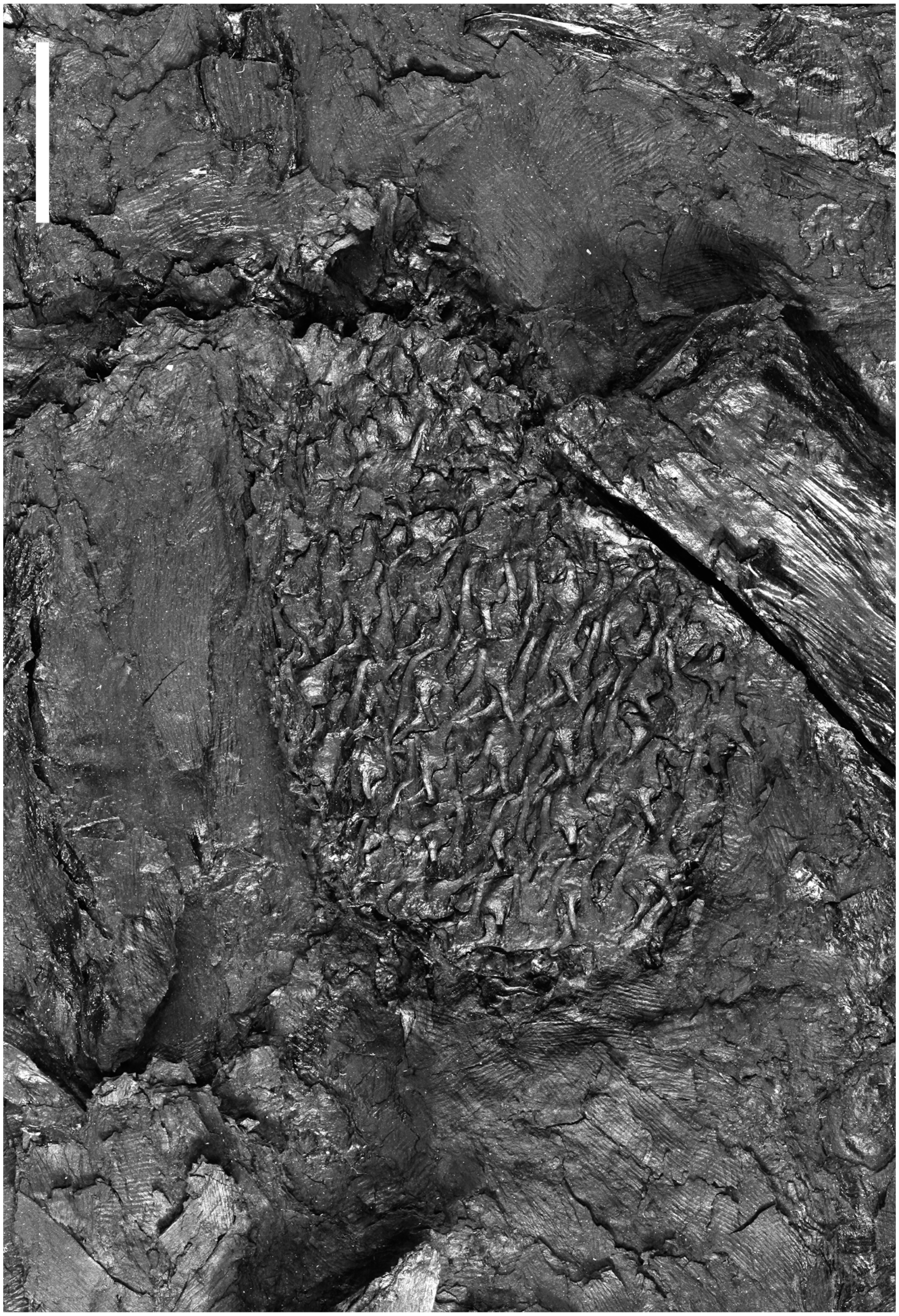

Articulated *Krassilovia mongolica* cone buried in a mat of *Podozamites harrisii* leaves. Scale bar = 5 mm.

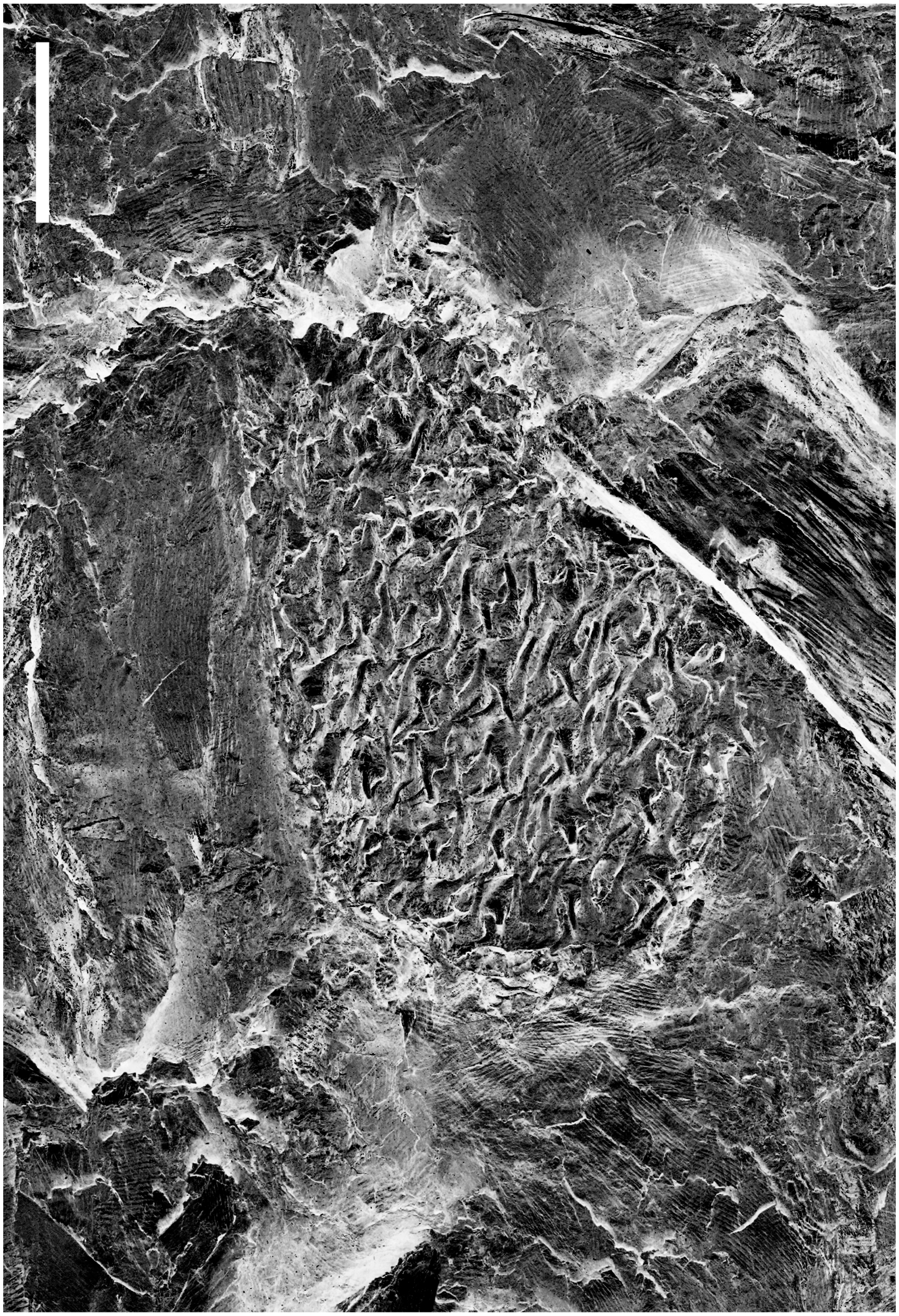

Inverted color image highlighting details of articulated *Krassilovia mongolica* cone buried in a mat of *Podozamites harrisii* leaves. Scale bar = 5 mm.
