# Supplementary material for: Reconstructing Krassilovia mongolica supports recognition of a new and unusual group of Mesozoic conifers
Source: PLoS One. 2020 Jan 15;15(1):e0226779. doi: 10.1371/journal.pone.0226779 (PMC6961850; doi:10.1371/journal.pone.0226779)
Supplement: S4 Appendix — (PDF) [file pone.0226779.s004.pdf]

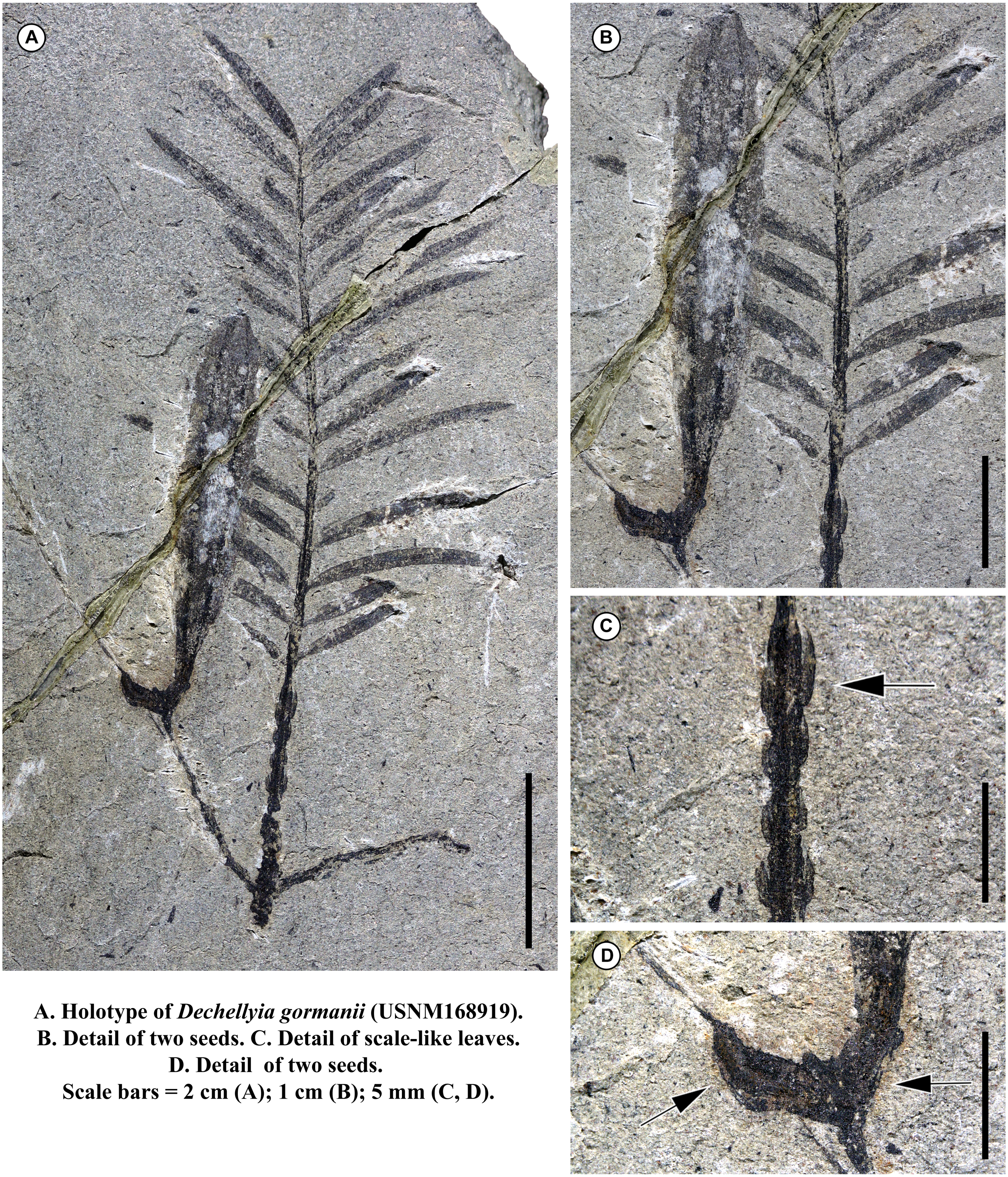

A. Holotype of *Dechellyia gormanii* (USNM168919).
B. Detail of two seeds. C. Detail of scale-like leaves.
D. Detail of two seeds.
Scale bars = 2 cm (A); 1 cm (B); 5 mm (C, D).
