# Supplementary material for: Reconstructing Krassilovia mongolica supports recognition of a new and unusual group of Mesozoic conifers
Source: PLoS One. 2020 Jan 15;15(1):e0226779. doi: 10.1371/journal.pone.0226779 (PMC6961850; doi:10.1371/journal.pone.0226779)

Full tree from Figure 5B

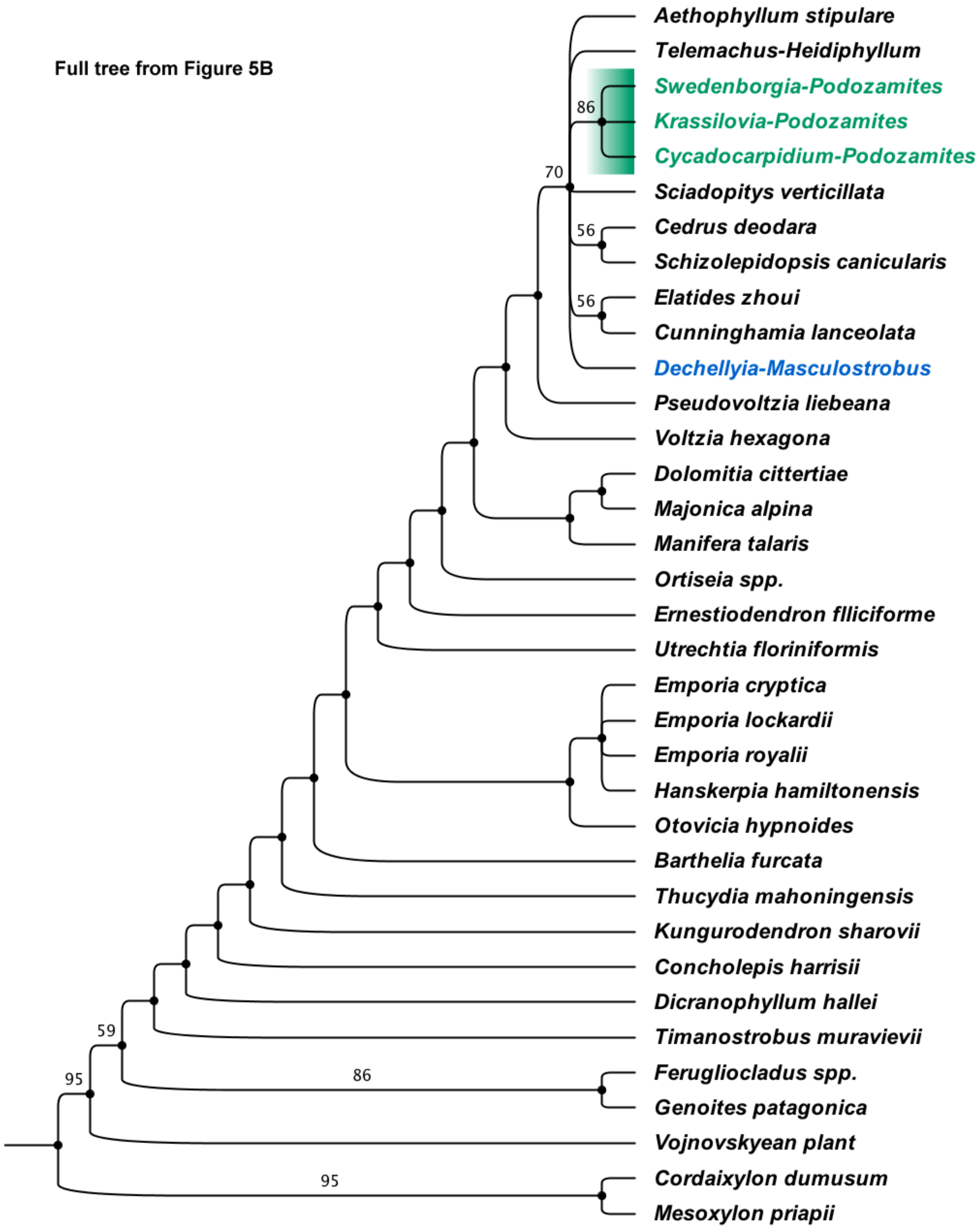

Analysis excluding living conifers

This analysis resulted in 25 most parsimonious trees (length 186 steps, [CI] 0.414, [RI] 0.691, and [RC] 0.286)

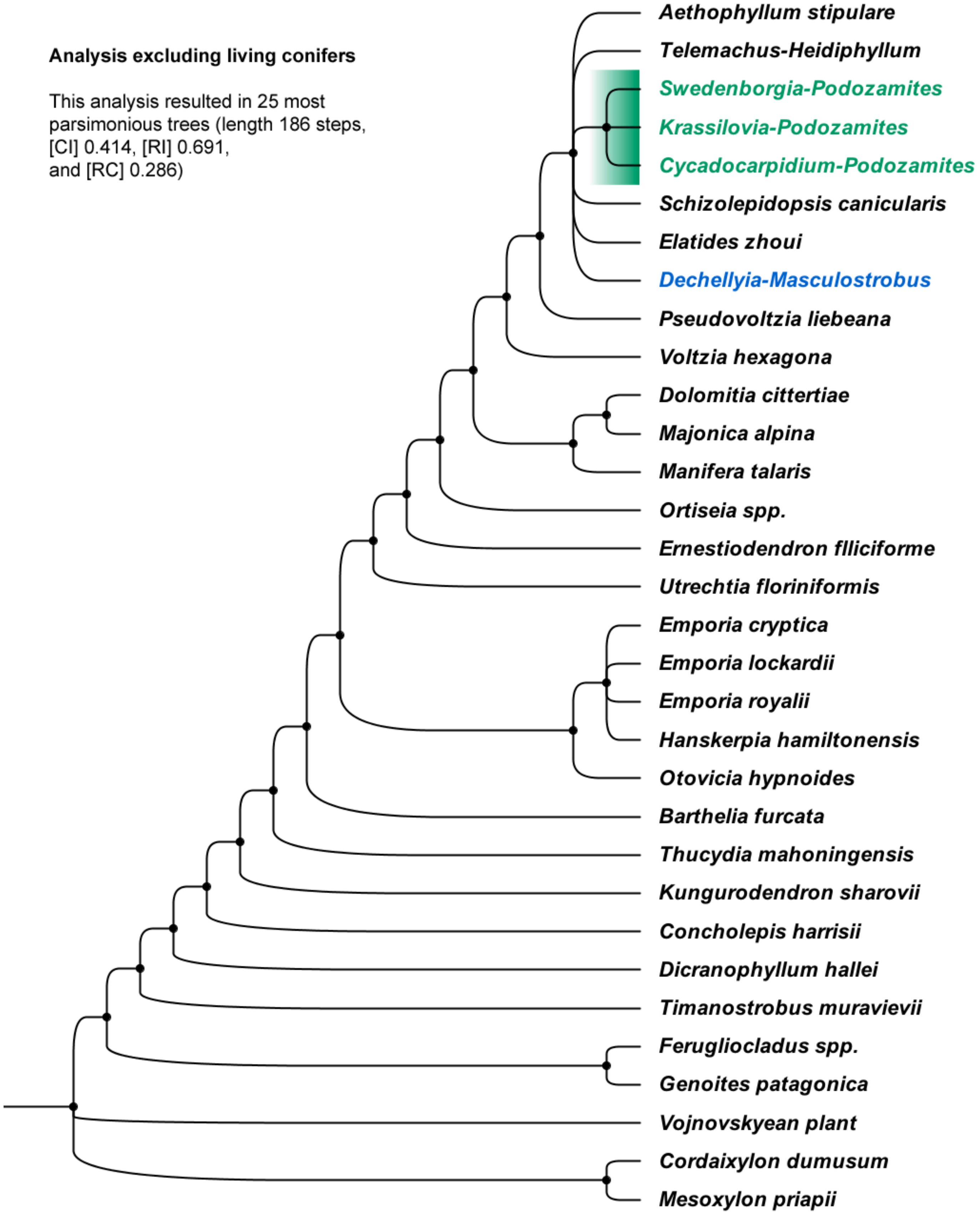

Analysis excluding living conifers, *Elatides*, and *Schizolepidopsis*

This analysis resulted in 5 most parsimonious trees (length 174 steps, [CI] 0.437, [RI] 0.692, and [RC] 0.302)

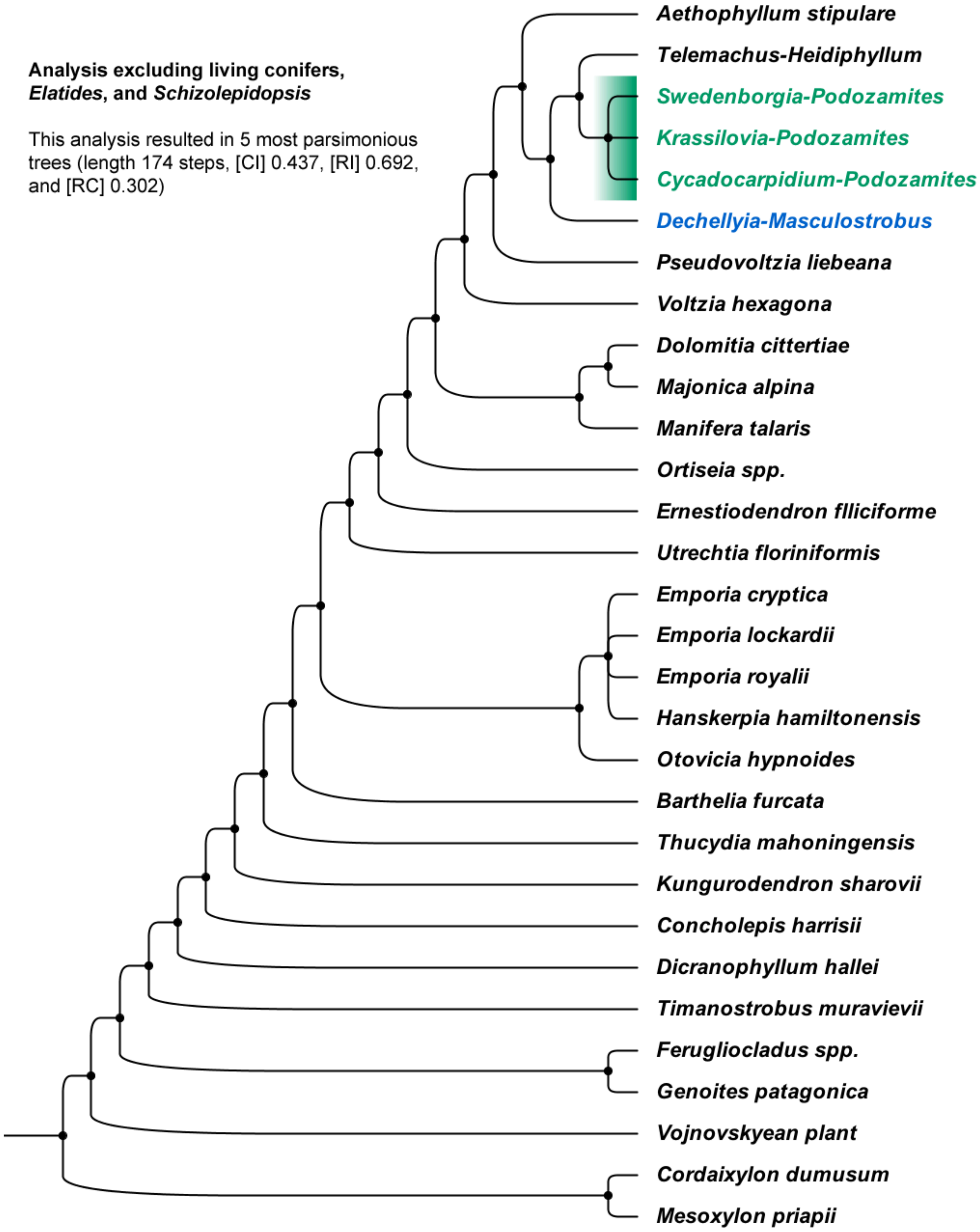

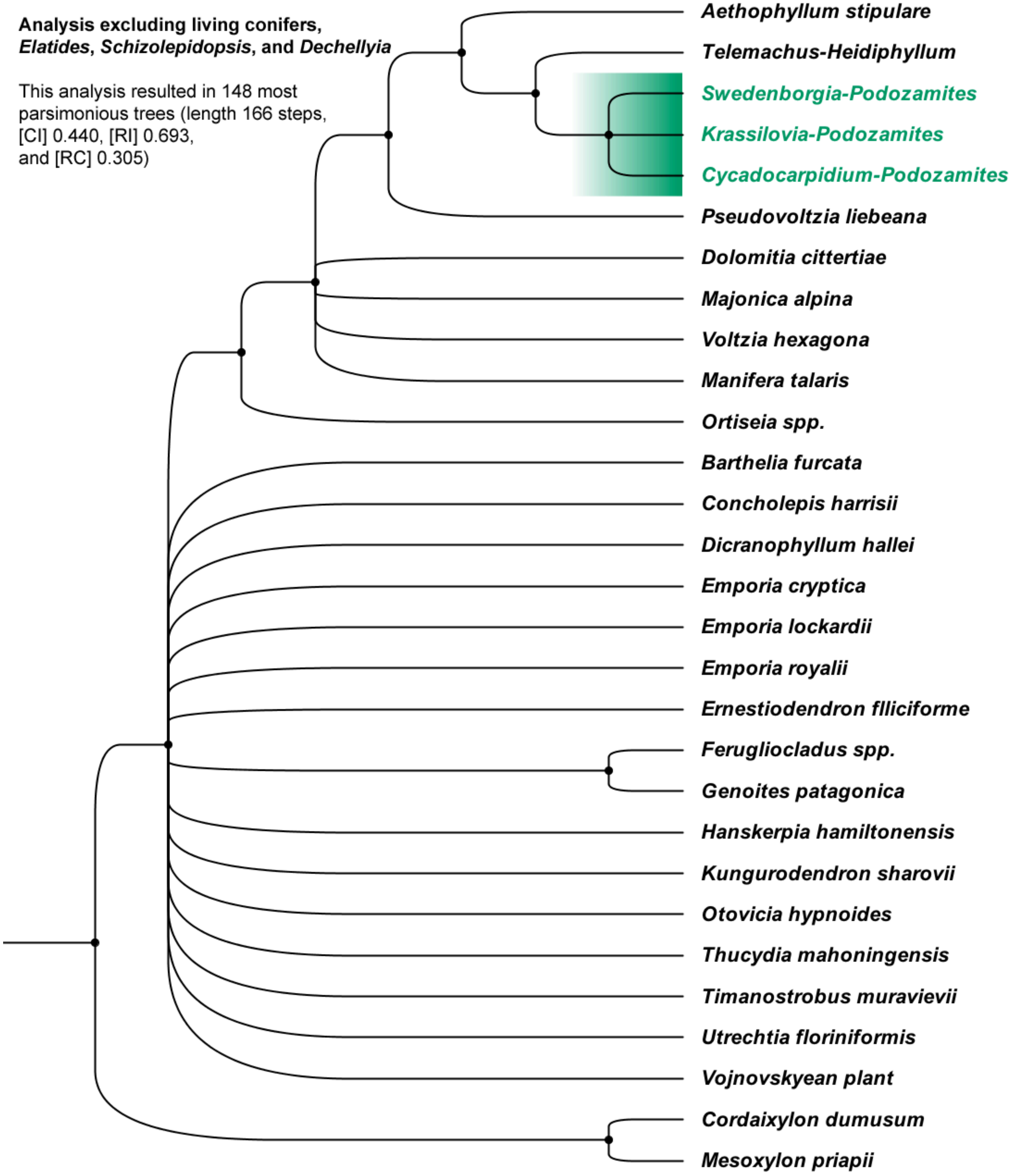
Analysis excluding living conifers, *Elatides*, *Schizolepidopsis*, and *Dechellyia*
This analysis resulted in 148 most parsimonious trees (length 166 steps, [CI] 0.440, [RI] 0.693, and [RC] 0.305)
Aethophyllum stipulare
Telemachus-Heidiphyllum
Swedenborgia-Podozamites
Krassilovia-Podozamites
Cycadocarpidium-Podozamites
Pseudovoltzia liebeana
Dolomitia cittertiae
Majonica alpina
Voltzia hexagona
Manifera talaris
Ortiseia spp.
Barthelia furcata
Concholepis harrisii
Dicranophyllum hallei
Emporia cryptica
Emporia lockardii
Emporia royalii
Ernestiodendron flliciforme
Ferugliocladus spp.
Genoites patagonica
Hanskerpia hamiltonensis
Kungurodendron sharovii
Otovicia hypnoides
Thucydia mahoningensis
Timanostrobus muravievii
Utrechtia floriniformis
Vojnovskyean plant
Cordaixylon dumusum
Mesoxylon priapii

Analysis including living conifers,
***Elatides*, *Schizolepidopsis,* and two Cheirolepidiaceae**
fossils: *Frenelopsis ramosissima* &
Pararaucaria taquetrensis.
This matrix includes 52 morphological characters.

This analysis resulted in 150 most parsimonious trees (length 202 steps, [CI] 0.396, [RI] 0.711, and [RC] 0.282)

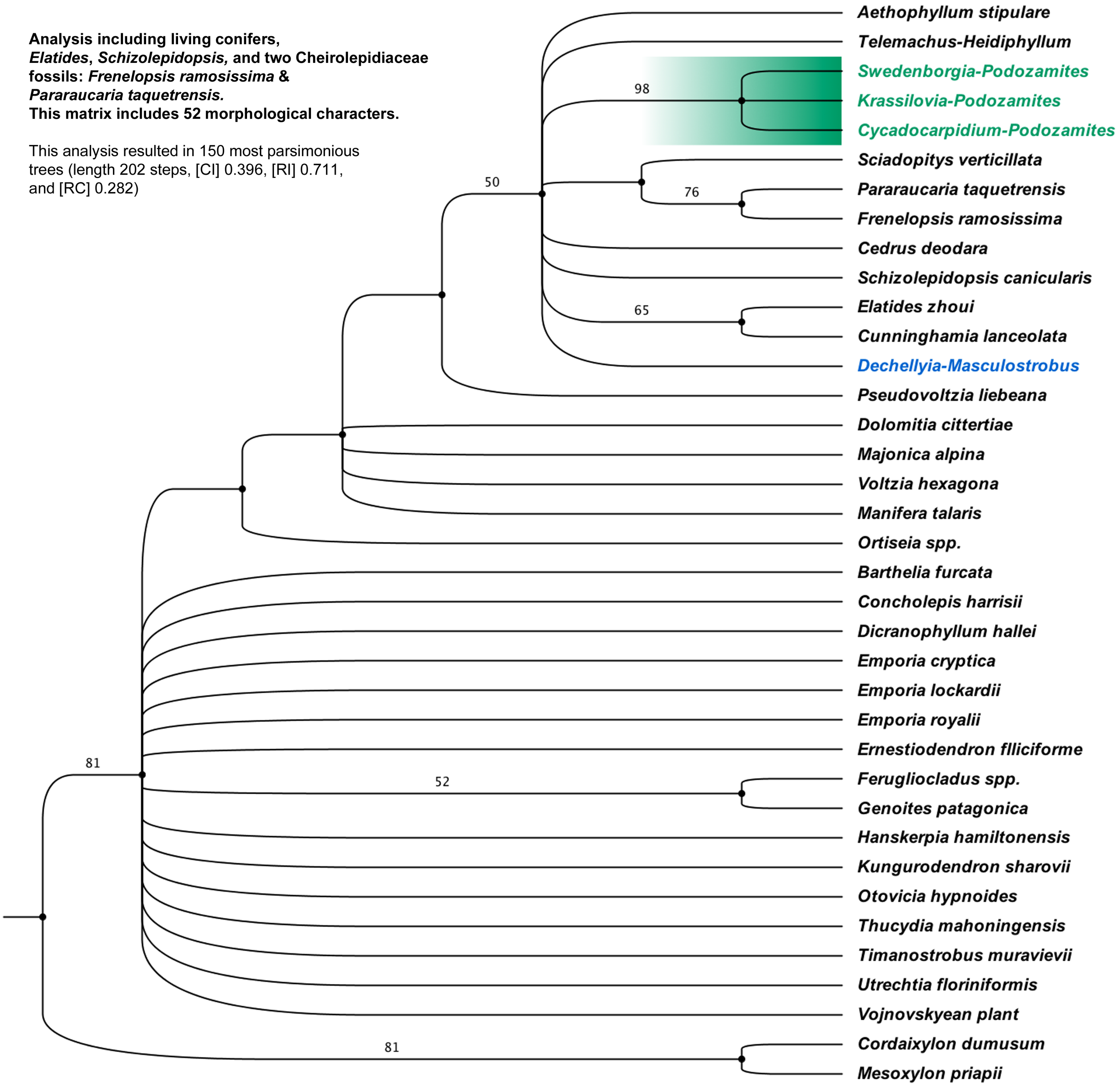

Analysis including living conifers, *Lebowskia grandifolia, Elatides*, *Schizolepidopsis,* and two Cheirolepidiaceae fossils: *Frenelopsis ramosissima* & *Pararaucaria taquetrensis.*
This matrix includes 52 morphological characters.

This analysis resulted in 48 most parsimonious trees (length 204 steps, [CI] 0.392, [RI] 0.712, and [RC] 0.279)

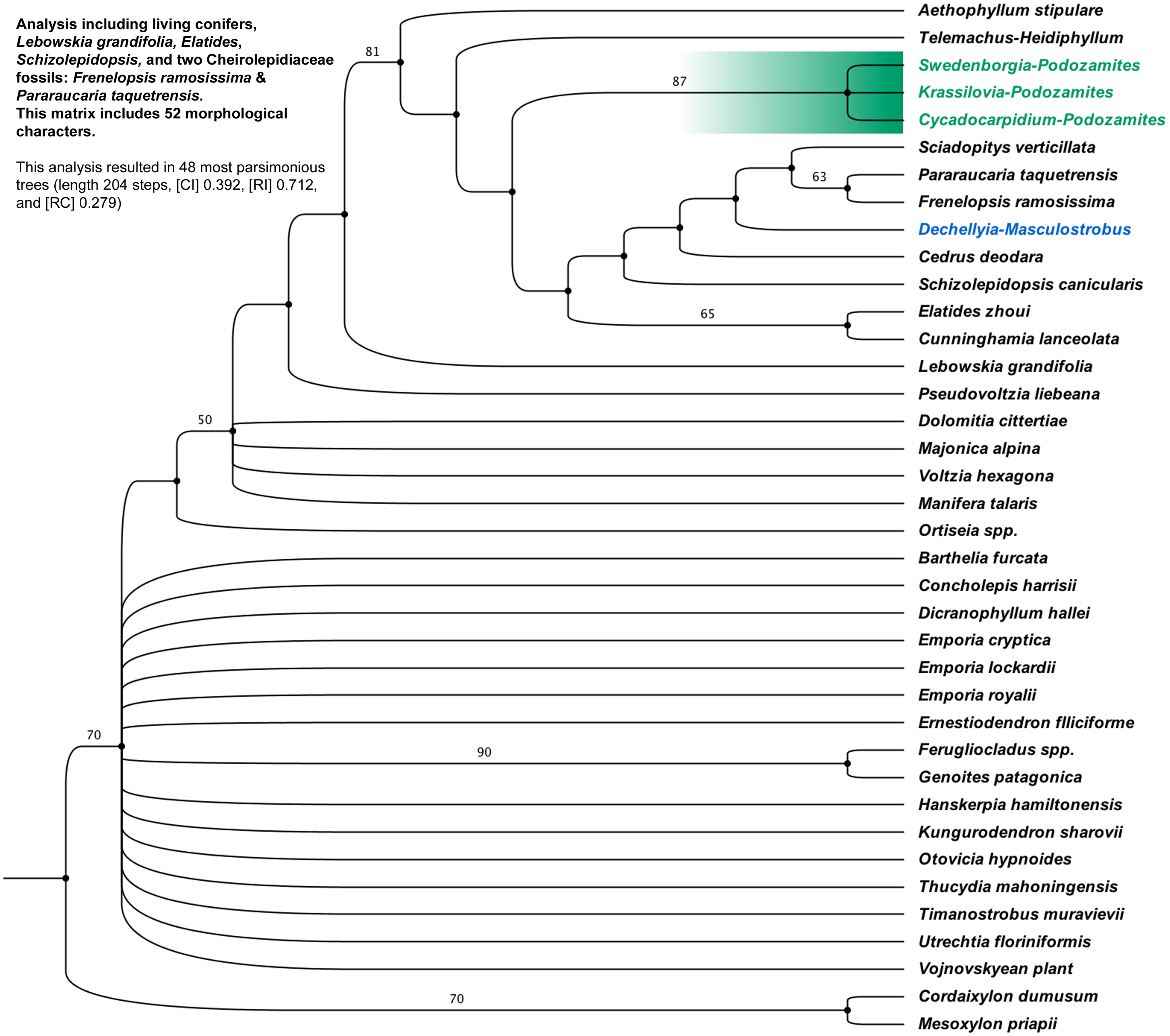

Supplement: S5 Appendix — (PDF) [file pone.0226779.s005.pdf]
