# Supplementary material for: Reconstructing Krassilovia mongolica supports recognition of a new and unusual group of Mesozoic conifers
Source: PLoS One. 2020 Jan 15;15(1):e0226779. doi: 10.1371/journal.pone.0226779 (PMC6961850; doi:10.1371/journal.pone.0226779)
Supplement: S6 Appendix — (PDF) [file pone.0226779.s006.pdf]

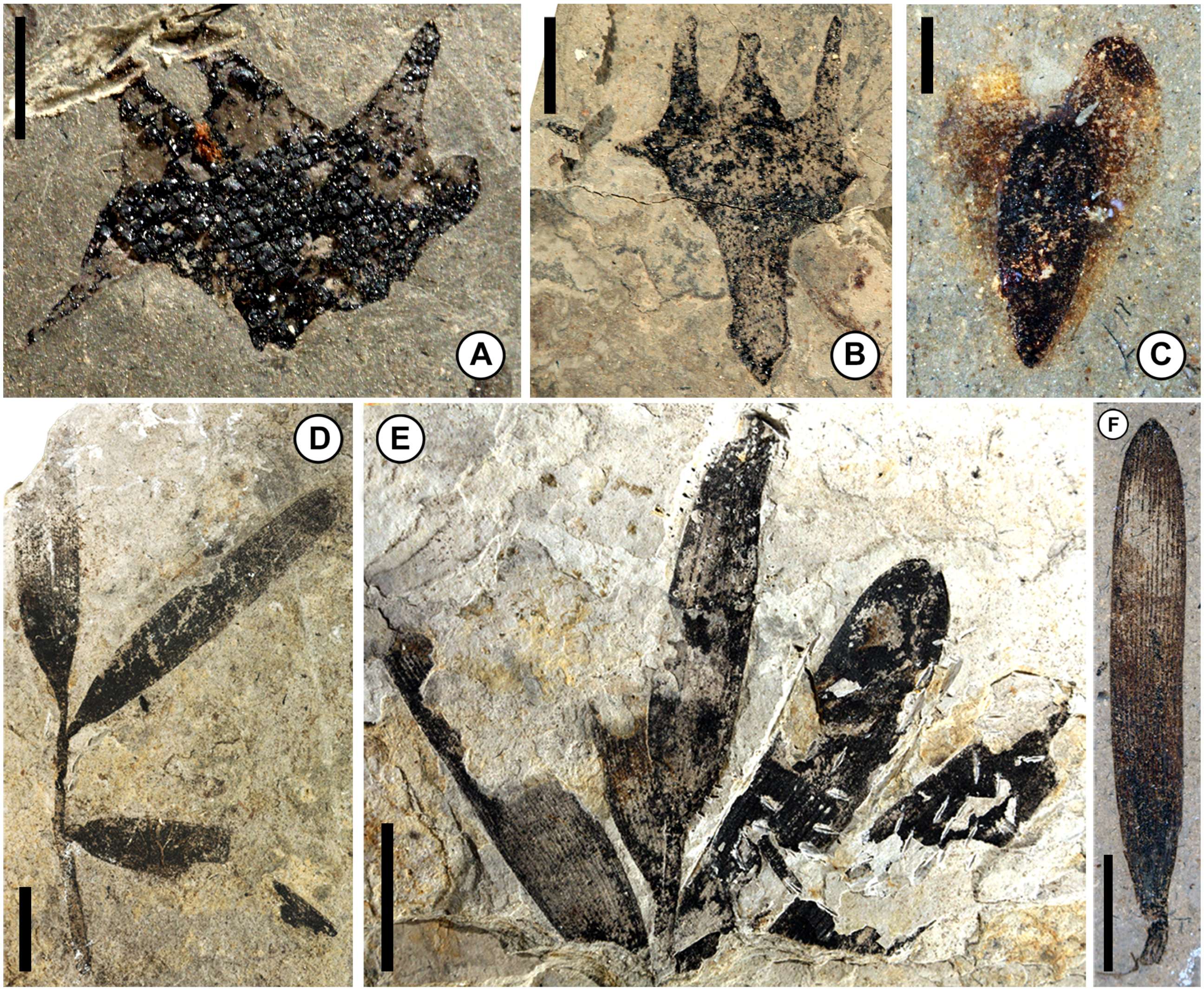

Additional specimens of the *Krassilovia* plant from Shine Khudag.(A, B) Bract-scale complexes. (C). Winged seed. (D, E) Attached shoots of *Podozamites harrisii* . (F) Isolated *P. harrisii* leaf. Scale bars = 1 cm (D, E); 5 mm (F); 2 mm (A); 1 mm (B, C).
